# Atlantic Salmon (*Salmo salar*) Performance Fed Low Trophic Ingredients in a Fish Meal and Fish Oil Free Diet

**DOI:** 10.3389/fphys.2022.884740

**Published:** 2022-06-08

**Authors:** K. Kousoulaki, L. Sveen, F. Norén, Å. Espmark

**Affiliations:** ^1^ Department of Nutrition and Feed Technology, Nofima, Tromsø, Norway; ^2^ Department of Fish Health, Nofima, Tromsø, Norway; ^3^ Marine Feed AB, Stenungsund, Sweden; ^4^ Department of Aquaculture Production Biology, Nofima, Tromsø, Norway

**Keywords:** Atlantic salmon, black soldier fly larvae, microalgae, tunicates, *Schizochytrium sp.*, low trophic level feed ingredients

## Abstract

To evolve fish farming in an eco-efficient way, feed production must become less dependent on forage fish-based ingredients and make more use of low trophic level organisms, including microalgae, higher plants, as filter feeding organisms and other ingredients with low competition to established food value chains. Diets nearly free of fish meal and fish oil are not a novelty but are often composed of complex mixtures, containing supplements to meet the farmed animal’s nutritional requirements. Sustaining a growing aquaculture production, maintaining at the same time fish health, welfare, and profitability, and meeting strict environmental and food safety demands, is challenging and requires new technologies, great investments, and more knowledge. A benchmarking feeding trial was performed to demonstrate the main effects of four low trophic raw materials on Atlantic salmon (*Salmo salar*) growth, metabolism, skin health and fillet quality. To this end, a diet was produced to contain commercially relevant levels of fresh high quality organic FM and FO and was used as a control in the trial (FMFO). Heterotrophically produced *Schizochytrium limacinum* biomass was used to replace organic FO (HM diet). Spray dried cell wall disrupted biomass of the phototrophically cultured diatom *Phaeodactylum tricornutum* replaced partly FM and FO (PM diet). Black soldier fly (*Hermetia illucens*) larvae meal and tunicate (*Ciona intestinalis*) meal, were used to produce the diets BSFL and TM, respectively, replacing large parts of FM as compared to the FMFO. A fifth test diet was produced combining all test raw materials and removing all FM and FO (0FM0FO diet). All test ingredients were well accepted sustaining high growth rates (TGC values near 4) and feed efficiency (FCR values below 0.9) in salmon showing good gut health and normal metabolic responses. However, none of the treatments reached the growth performance of FMFO. Additional differences between test and control treatments were identified in dietary nutrient apparent digestibility, fish biometrics, blood metabolites and fillet and skin composition. Extensive raw material and dietary chemical characterisation was performed to provide insight on potential shortcomings in the novel low trophic level ingredients which can possibly be overcome combining complementary raw materials.

## Introduction

A plethora of different raw materials are currently being explored as candidates to replace finite marine resources (Heal and Schlenker 2009; Beal et al., 2018) used to produce fish meal (FM) and fish oil (FO) for aquaculture diets. Contemporary Atlantic salmon farming trends focus on using low trophic level organisms as feed ingredients, preferably produced locally, with potential to achieve high production volumes, with low environmental effects, low carbon footprint and based on circularity principles (e.g., PhI et al., 2020). Moreover, the increasing consumer awareness on food safety and sustainability, has introduced a steady demand for organic products in the market (Polymeros et al., 2014; Lernoud and Willer, 2018), adding to the complexity of successful and sustainable fish farming growth strategies. Several of the so far novel feed raw materials considered have different nutritional and technical performance from FM, FO, and high protein higher plant meals. This is an additional practical challenge that the sector is facing in replacing established value chains with new local ones, at adequate amounts and competitive costs. New low trophic raw materials often contain high levels of complex carbohydrates, such as those present in rigid bacterial, yeast and algal cell wall structures (e.g., Harrison et al., 1991; Safi et al., 2013; Wu et al., 2015). Ingredients produced from arthropods contain significant exoskeleton chitin amounts shown to limit performance in fish species like rainbow trout (Lindsay et al., 1984) whereas being well digested by others, like cod (Toppe et al., 2006) possessing lower gastric pH (Danulat, 1987), and high chitinolytic enzyme activity both in the stomach, and in the intestinal contents (Danulat and Kausch, 1984). *Schizochytrium limacinum* (*S. limacinum*) is a DHA rich fungal protist, often referred to as microalgae or microalgae - like organism, known to store fully saturated triglycerides, such as tripalmitin, whereas only one or two but not 3 saturated fatty acids are present in FO triglycerides (Bogevik et al., 2018). Fully saturated fatty acids are less digestible in fish farmed in low water temperatures, such as Atlantic salmon (Kousoulaki et al., 2016). New ingredients may also have very different rheological properties affecting the technical quality of extruded feeds (Samuelsen et al., 2018; 2022). Thus, considering new ingredients for aquatic diets, their nutritional value, best use practices and safety must be thoroughly studied before establishing their commercial production and use.

Microalgae, being in the bottom of the trophic pyramid, and primary producers of essential nutrients (Ahlgren et al., 1992; Helliwell et al., 2016; Duncan and Petrou, 2022) are popular candidates as new feed ingredients and supplements (Beal et al., 2018; Tocher et al., 2019). Phototrophic microalgae and microalgae - like heterotrophic biomass can be produced practically anywhere in closed photobioreactors (Chen et al., 2011) or dark fermentation containers (Turon et al., 2016), respectively. They require minimal amounts of fresh water and can be produced in conjunction with other industrial food production activities, using sidestream biomasses as cultivation inputs, as for instance CO_2_, waste heat and organic by products (Ren et al., 2018). Not surprisingly, entrepreneurship on the development of microalgal based industries is in focus with heterotrophic production showing the lowest environmental impacts, mainly due to high production densities and favourable oil content and quality (Barba et al., 2014; Lu et al., 2021). The chemical composition of the phototrophic microalgal strains varies considerably in terms of total protein, carbohydrate, and lipids, but their amino acid profile is similar to FM (Tibbetts, 2018). In the present study we used cell wall disrupted diatom biomass of the species *Phaeodactylum tricornutum* (*P. tricornutum*) as in whole biomass the presence of intact rigid cell walls may compromise the bioavailability of micro algal nutrients in carnivorous fish (Sørensen et al., 2016). Heterotrophically produced *Schizochytrium* sp. biomass (Tables 1–3) contains higher levels of lipids and yields good performance results in Atlantic salmon (*Salmo salar*) at low and moderately high dietary levels (Kousoulaki et al., 2015), providing benefits also in low FM diets (Kousoulaki et al., 2016). Moreover, in a life - long feeding trial in Atlantic salmon, replacement of FO by heterotrophically produced *S. limacinum* biomass yielded encouraging results including better growth, higher flesh eicosapentaenoic acid (EPA) and docosahexaenoic acid (DHA) levels and improved pigmentation (Kousoulaki et al., 2020b).

**TABLE 1:** Formulation of the experimental diets used in the current trial. Raw material levels are given in g 100^−1^ of total raw material mix.

	FMFO	TM	BSFL	PM	HM	0FM0FO
Fish meal	25.00	13.60	8.01	20.30	24.50	
Insect meal			20			20
Tunicate meal		20				20
*Schizochytrium limacinum*					7.7	7.7
*Phaeodactylum tricornutum*				7.5		7.5
Wheat gluten	16.92	18.49	16.51	15.95	15.16	14.77
Horse beans	16.00	14.00	16.00	16.00	16.00	2.00
Wheat meal	16.10	5.00	11.40	13.00	13.30	5.00
Krill hydrolysate (60% dry matter)		2.00	2.00	2.00	2.00	2.00
Fish oil	10.10	9.20	11.80	9.10		
Rapeseed oil	5.75	5.95	4.75	5.43	9.60	9.50
Linseed oil	0.75	1.50	0.60	1.00	2.70	2.50
Rapeseed lecithin	1.00	1.70	0.85	1.20	0.80	1.30
Vitamin premix^a^	2.00	2.00	2.00	2.00	2.00	2.00
Mineral premix	0.50	0.50	0.50	0.50	0.50	0.50
NaH2PO4	2.20	2.40	2.00	2.30	2.20	1.75
Biomoss	0.50	0.50	0.50	0.50	0.50	0.50
Choline chloride	0.50	0.50	0.50	0.50	0.50	0.50
Cholesterol	0.50	0.50	0.50	0.50	0.50	0.50
Lysine	0.67	0.70	0.68	0.70	0.62	0.73
Methionine	0.24	0.29	0.35	0.25	0.23	0.34
Threonine	0.39	0.29	0.38	0.36	0.33	0.20
Histidine	0.77	0.77	0.56	0.80	0.75	0.60
Carop. Pink (35%)	0.05	0.05	0.05	0.05	0.05	0.05
Stay-C	0.05	0.05	0.05	0.05	0.05	0.05
Yttrium oxide	0.01	0.01	0.01	0.01	0.01	0.01
Sum	100	100	100	100	100	100
						
Analysed composition in DM						
Protein%	44.6	43.9	43.8	43.8	44.6	43.7
Lipid%	23.6	23.9	24.3	24.6	24.2	25.1
Ash%	6.5	11.1	5.6	7.4	6.9	11.9
Total P%	1.29	1.09	1.09	1.31	1.31	0.97
Soluble P%	0.84	0.83	0.87	0.87	0.81	0.83
Starch%	20	12.6	16.7	18.2	17	7.4
Combustion value kJ g^−1^	23.84	22.79	24.31	23.64	23.69	22.78
Yttrium mg kg^−1^	82.99	88.71	78.94	86.93	83.15	85.44
						
Pellet technical quality^b^						
Water stability index% (WSI)	79.48 ^bc^ ±0.03	74.99^b^ ± 3	83.06^c^ ±0.24	79.72 ^bc^ ±1.02	81.01 ^bc^ ±0.1	17.58^a^ ±3.79

a Provides vitamins to a final feed concentration of 2000 IU, vitamin A, 2500 IU, vitamin D3, 200 mg/kg vitamin E, 20 mg/kg vitamin K3, 20 mg/kg vitamin B1, 30 mg/kg vitamin B2, 30 mg/kg vitamin B6 pyridoxin-HCl, 25 mg/kg vitamin B6 pyridoxin, 0.05 mg/kg vitamin B12, 10 mg/kg folic acid, 60 mg/kg Ca-d-pantothenic acid, 200 mg/kg niacin, and 1 mg/kg vitamin H (biotin).

b Water stability index values with different small superscript letter are significantly different (*p* = 0.000) following Tukey post hoc test. Each value is a mean of 3 replicate analyses.

**TABLE 2:** Fatty acid profile and lipid oxidation status of the experimental diets used in the current trial.

		FMFO	TM	BSFM	PM	HM	0FM0FO
14:0	g/100 g B&D extract	3.70	3.30	4.40	3.60	1.30	1.60
16:0	g/100 g B&D extract	8.50	8.10	9.00	8.60	13.80	14.50
18:0	g/100 g B&D extract	1.30	1.30	1.20	1.30	1.80	1.70
20:0	g/100 g B&D extract	0.30	0.30	0.20	0.30	0.30	0.30
22:0	g/100 g B&D extract	0.10	0.10	0.10	0.10	0.20	0.20
**Saturated fatty acids**	**g/100 g B&D extract**	**13.90**	**13.10**	**14.90**	**13.90**	**17.40**	**18.30**
16:1 n-7	g/100 g B&D extract	2.00	1.90	2.20	3.00	0.50	1.60
18:1 (n-9)±(n-7)±(n-5)	g/100 g B&D extract	24.60	26.10	22.70	24.80	32.90	29.60
20:1 (n-9)±(n-7)	g/100 g B&D extract	7.30	6.50	7.50	6.80	1.60	0.70
22:1 (n-11)±(n-9)±(n-7)	g/100 g B&D extract	10.90	9.40	11.40	10.00	1.40	0.20
24:1 n-9	g/100 g B&D extract	0.40	0.40	0.40	0.40	0.10	<0.1
**Monounsaturated fatty acids**	**g/100 g B&D extract**	**45.20**	**44.30**	**44.20**	**45.00**	**36.50**	**32.10**
16:2 n-4	g/100 g B&D extract	0.20	0.10	0.20	0.30	<0.1	0.20
16:3 n-4	g/100 g B&D extract	0.10	0.10	0.10	0.30	<0.1	0.20
18:2 n-6	g/100 g B&D extract	9.80	10.50	9.80	10.00	13.80	13.50
18:3 n-6	g/100 g B&D extract	0.10	0.10	0.10	0.10	<0.1	<0.1
20:2 n-6	g/100 g B&D extract	0.10	0.10	0.10	0.10	0.10	0.10
20:3 n-6	g/100 g B&D extract	<0.1	<0.1	<0.1	<0.1	<0.1	<0.1
20:4 n-6	g/100 g B&D extract	0.10	0.10	0.10	0.20	0.10	0.10
22:4 n-6	g/100 g B&D extract	<0.1	<0.1	<0.1	<0.1	<0.1	<0.1
**PUFA (n-6) fatty acids**	**g/100 g B&D extract**	**10.10**	**10.80**	**10.10**	**10.40**	**14.00**	**13.70**
18:3 n-3	g/100 g B&D extract	4.80	6.60	4.30	5.50	11.00	9.10
18:4 n-3	g/100 g B&D extract	1.30	1.10	1.40	1.20	0.20	0.10
20:3 n-3	g/100 g B&D extract	0.10	<0.1	<0.1	<0.1	<0.1	<0.1
20:4 n-3	g/100 g B&D extract	0.30	0.20	0.30	0.20	0.10	0.10
20:5 n-3 (EPA)	g/100 g B&D extract	3.00	2.50	3.00	3.40	0.70	0.90
21:5 n-3	g/100 g B&D extract	0.10	0.10	0.10	0.10	<0.1	<0.1
22:5 n-3	g/100 g B&D extract	0.50	0.50	0.50	0.50	0.30	0.20
22:6 n-3 (DHA)	g/100 g B&D extract	3.90	3.10	3.50	3.60	5.90	4.90
**PUFA (n-3) fatty acids**	**g/100 g B&D extract**	**14.00**	**14.10**	**13.10**	**14.50**	**18.20**	**15.30**
Omega-6/omega-3 ratio		0.73	0.77	0.78	0.71	0.76	0.90
EPA ± DHA	g/100 g B&D extract	6.90	5.60	6.50	7.00	6.60	5.80
Identified fatty acids	g/100 g B&D extract	83.50	82.50	82.60	84.40	86.10	79.80
Unidentified fatty acids	g/100 g B&D extract	2.60	2.40	5.50	2.70	2.30	5.60
Free fatty acids	% in B&D extract	6.40		6.10	10.10	5.70	8.80
Anisidine value		12.00		1.70	49.00	22.00	62.00
Peroxide value	meq peroxide/kg fat	15.00		12.00	22.00	11.00	21.00

**TABLE 3:** Protein bound amino acid profile of the experimental diets used in the current trial. Tryptophane and cysteine analyses were not performed.

		FMFO	TM	BSFM	PM	HM	0FM0FO
Aspartic acid	%	2.50	2.50	2.40	2.50	2.60	2.70
Glutamic acid	%	9.20	9.00	8.40	8.70	8.60	7.80
Hydroxyproline	%	0.17	0.14	0.13	0.17	0.19	<0.10
Serine	%	1.70	1.70	1.50	1.60	1.70	1.70
Glycine	%	1.90	1.80	1.70	1.80	1.80	1.80
Histidine	%	1.30	1.30	0.84	1.30	1.30	1.20
Arginine	%	2.00	1.90	1.80	2.00	2.00	1.80
Threonine	%	1.60	1.50	1.40	1.60	1.60	1.50
Alanine	%	1.70	1.50	1.70	1.70	1.70	1.70
Proline	%	2.80	2.80	2.80	2.80	2.60	2.80
Tyrosine	%	1.10	1.20	1.40	0.98	0.99	1.50
Valine	%	1.70	1.70	1.70	1.60	1.60	1.80
Methionine	%	0.98	0.97	0.94	0.93	0.94	0.97
Isoleucine	%	1.50	1.50	1.40	1.50	1.50	1.50
Leucine	%	2.80	2.70	2.70	2.70	2.70	2.60
Phenylalanine	%	1.60	1.60	1.60	1.60	1.60	1.60
Lysine	%	2.50	2.30	2.20	2.40	2.50	2.20
Total amino acids	%	35.2	35.9	35.9	34.6	36.1	37.1
Total EAA	%	15.98	15.47	14.58	15.63	15.74	15.17

Tunicates belong to a group of filter-feeding sea invertebrates (ascidians, also known as sea squirts) growing in shallow ocean waters worldwide. They trap prey particles by pumping water through the oral siphon and thus functions as a biological water filtration system that can remove microalgae from eutrophic plankton - rich waters. Though tunicates have historically not been viewed as a valuable fish feed source, in the recent years, the interest in this organism has grown. In the present work we tested a tunicate meal produced by the tunicate species *Ciona intestinalis*, produced by Marine Feed AB. The tunics (45% of total animal dry weight) of *Ciona intestinalis*, composed mainly of valuable animal cellulose (Angles and Dufresne, 2000; Mathew and Dufresne, 2002), protein, and ash, act as a skeletal structure, whereas the inner animal (55% of total animal weight) consists mainly of protein and *ω* - 3 fatty acids and may be used as feed ingredients (Kousoulaki et al., 2020a).

Insects are part of salmon parr’s natural pray (Henry et al., 2015). In a study in the Louvenga River, Kola Peninsula, Russia, aerial insects represented 24% of the food items found in wild Atlantic salmon parr together with aquatic insect larvae and pupae that represented 68.2% of their diet (Orlov et al., 2006). In the same study, the feeding behaviour of farmed Atlantic salmon parr that were released in the same river differed from that of the wild fish with aquatic insect larvae and pupae representing lower (49%) and terrestrial insects, higher (32%) proportion of their diet. Protein rich insect meals can be produced converting low value feedstuffs, including co - products of the agricultural industry, into high value protein and oils in a circular economy manner. Replacing FM and FO in aquafeeds by insect - based ingredients can reduce the economic fish - in - fish - out value and land use of the aquaculture and provide in the future a sustainable solution for further growth of the sector (Quang Tran et al., 2022). Insect meals can vary largely in nutrient composition, e.g., ranging between 9.3 and 76% in protein and 7.9–40% in fat content (as reviewed by Nogales-Mérida et al., 2019). Recent studies on the use of insect meals in diets for aquaculture fish species including Atlantic salmon, have shown encouraging results replacing up to 100% of dietary FM with some types of insect meals and revealed shortcomings of other (e.g., Lock et al., 2016). Atlantic salmon performed equally when fed a FM - based control diet and diets where FM was substituted by black soldier fly meal at lower dietary inclusion levels (8–16% in the diet) with a trend to deteriorating performance at increasing black soldier fly larvae dietary inclusion level (32%) (Weththasinghe et al., 2019). Belghit et al. (2018) saw lower protein digestibility followed by non - statistically significant growth reduction and elevated hepatosomatic index in Atlantic salmon fed 60% of black soldier fly larvae meal in the diet.

In the present study we benchmarked four different low trophic level organism - based ingredients, namely, tunicate meal, insect meal, phototrophic microalgae biomass and heterotrophically produced *S. limacinum* biomass against a high - quality organic FM and FO diet. Our aim was to identify opportunities and limitations in using these raw materials in salmon diets alone or combined in a FM and FO free diet, in terms of nutrient availability, and effects on general fish performance, metabolism, skin health and nutritional quality of flesh.

## Materials and Methods

### Test Ingredient Sourcing

Freshly produced organic FM, made from herring by-product (51%), Norway Pout (25%), sprat (18%) and blue whiting (6%), and organic FO produced from Norwegian spring spawning herring by product, Norwegian spring spawning herring, herring by-product and herring, were provided by Pelagia factories in Egersund (Norway) and Måløy (Norway), respectively. Organic FM and FO must be stabilised with different tocopherol extracts (E 306) and its origin must be either from offal from organic aquaculture or from cuts from fish already caught for human consumption in sustainable fisheries, the latter was the case in this study. The *P. tricornutum* (ND58; Prestegard et al., 2009) biomass used in this trial was provided by the NORCE, Bergen, Norway, and produced at the National Algae pilot Mongstad (NAM; Mongstad, Norway) in a fed-batch process using four 800 L photobioreactors (GemTube MK2-750 from LGem, Rotterdam, Netherlands). The *P. tricornutum* culture was harvested and concentrated by centrifugation (Evodos 50, Evodos b. v) and the concentrated paste (dry matter (DM) content 22–35%) was delivered vacuum packed and frozen to Nofima in Bergen, Norway, and kept at −23°C until further use. The biomass was thawed by heating to 60°C, disrupted to an approximate 92% cell wall disruption degree (estimated microscopically), and spray dried to a fine powder. Cell wall disruption was performed using a Dyno-Mill Multi Lab (Willy A. Bachofen, Muttenz, Switzerland) at 80% chamber filling rate in a 1.4 L milling chamber, using glass beads of 0.75 mm diameter. The mill was operated at 12 m s^−1^ tip speed (2,865 rpm). The biomass was diluted with tap water when necessary to approx. 22% dry matter and processed at an approximate flow rate of 7–9 kgh^−1^ and processing temperature of ca. 25–27°C. Spray dried *S. limacinum* biomass was provided by Alltech Inc. (Dunboyne, Ireland) and was the same batch as the one used in Kousoulaki et al. (2020b). Tunicate meal (*Ciona intestinalis*) was provided by Marine Feed AB (Stenungsund, Sweden) and black soldier fly larvae meal (*Hermetia illucens*) was supplied from INNOVAFEED (Gouzeaucourt, France). Krill hydrolysate was added as attractant in the test diets with lower FM or/and FO as compared to the high FM and FO diet used as control, with the aim to reduce the risk of obtaining physiological differences between the treatments due to low feed intake rates (Kousoulaki et al., 2013). The test raw materials were extensively characterised for their content in protein, lipids, water, ash, trace minerals (Cu, Fe, Mn, Zn and Se), total and soluble phosphorus (P), vitamins, total and free amino acid and fatty acid profile, lipid class composition, soluble protein level and soluble protein peptide size distribution, nucleotides, lipid oxidation and presence of undesirable compounds.

### Experimental Diets

A control diet was produced containing relatively high organic FM (25% in the diet) and FO (10% in the diet) levels (FMFO) (Table 1). Two more test diets were produced containing high inclusion levels of either black soldier fly larvae or tunicate heat and air-dried meals (BSFL and TM, respectively), partly substituting FM as compared to the FMFO diet. Two more test diets were produced containing heterotrophically produced *S. limacinum* spray dried biomass substituting FO (HM), or cell wall disrupted and spray dried *P. tricornutum* biomass partly substituting FM and FO (PM), as compared to the FMFO diet. A fifth test diet was produced combining all test ingredients at equal levels present in the respective single ingredient replacement test diets, substituting all dietary FM and FO (0FM0FO). The diets were balanced for protein, non-dispensable amino acids, EPA ± DHA and n-3/n-6 ratio, phospholipids, and soluble P, using wheat and horse beans, crystalline amino acids, plant oils, lecithin, and monosodium phosphate, respectively. The reasoning behind our test feed formulation choices was to use high levels of the test ingredients to be able to substitute FM and FO in the FO0FM0 diet with low risk of inducing negative health and production performance effects, and at the same time reveal potential positive or negative effects from the special micro and macro-elements that were not specially balanced for, and their in between interactions.

The experimental diets were produced at the Feed Technology Centre of Nofima in Bergen, Norway, in the same production series, using a Wenger TX-52 co-rotating twin-screw extruder with 150 kgh^−1^ capacity. The settings of the extruder were close to commercial i.e., the production can be scaled up to a feed factory, slightly adjusted during production from diet to diet. The screw configuration of the extruder was D, die opening 2.5 mm, knife speed varied between 1,000 and 1,450 rpm to yield pellets of similar length, SME was 6.2–6.9 kW, feed rate approx. 110–120 kg h^−1^, amount of steam added in the DDC conditioner was 11.7–12.3 kg h^−1^ and 0 h^−1^ in the extruder, water added in the DDC conditioner was 0.120–0.140 kg min^−1^ and in the extruder 0.080–0.160 kg min^−1^. The set temperatures in the extruder heads (H) 2-7 were H2: 83°C (achieved 75–90°C), H3: 110–120°C (achieved 100–112°C), H4: 120–130°C (achieved 88–109°C), H5: 115–140°C (not measured), H7: 70–80°C (achieved 71–80°C). The diets were analysed for water stability index (WSI) and their content in crude protein, crude lipid, gross energy, crude starch, phosphorus (P), yttrium, total amino acid, and fatty acid profile, vitamins and soluble peptide molecular size distribution (Tables 1–5).

**TABLE 4:** Vitamin content of experimental diets used in the current trial. Empty cells represent missing values.

		FMFO	TM	BSFM	PM	HM	0FM0FO
Vitamin A	mg/kg	4.19	1.87	3.87	3.49	2.27	0.62
Vitamin D3	mg/kg	0.191	0.137	0.2	0.177	0.0788	0.0601
Vitamin K1	ug/kg	149	172	121	132	189	
Vitamin C (ascorbyl-2-phosphate)	mg/kg	233	206	252	262	230	237
Vitamin C (ascorbic acid)	mg/kg	<5	<5	<5	<5	<5	
alpha Tocopherol	mg/kg	256	224	279	253	263	242
beta Tocopherol	mg/kg	<5	<5	<5	<5	<5	5.14
gamma Tocopherol	mg/kg	41.8	7.11	48.8	35.8	60.9	18.7
delta Tocopherol	mg/kg	8.11	<5	8.37	15.3	7.66	12.4
Vitamin E (sum tocopherols)	mg/kg	306	231	337	304	332	278

**TABLE 5:** Peptide size distribution of the water-soluble proteins in the current trial’s experimental diets. Values are given in% of water soluble peptides unless otherwise specified.

	FMFO	TM	BSFM	PM	HM	0FM0FO
Soluble protein% in sample	9.5	9.2	8.85	10.2	10.7	9.5
>20,000	1.2	1.2	1.0	0.8	0.8	0.8
15,000–20,000	2.7	2.7	2.5	2.1	2.1	1.9
10,000–15,000	6.8	7.0	6.5	5.2	5.3	4.9
8,000–10,000	3.8	3.5	4.4	2.9	3.0	3.2
6,000–8,000	4.7	4.1	5.6	3.6	3.7	4.0
4,000–8,000	6.2	5.3	6.3	5.0	5.2	4.4
2000–4,000	8.0	6.9	7.6	6.9	7.5	5.4
1,000–2000	4.7	5.2	5.5	5.2	5.7	5.0
500–1,000	3.1	4.7	4.8	4.3	4.6	5.4
200–500	4.5	6.8	7.1	5.9	3.2	8.5
<200	54.3	52.6	48.5	58.0	55.9	56.5

### Atlantic Salmon Smolt Feeding Trial

The experimental diets were fed to triplicate Atlantic salmon smolt groups of 50 fish per tank (500 L) in indoor tanks at Nofima facilities in Sunndalsøra, Norway. The experimental fish used were Atlantic salmon smolt of organic production provided by SalMar ASA (Frøya, Norway), with initial mean fish body weight 141.7 ± 5.6 g. The experimental tanks were equipped with continuous light and flow through sea water systems using UV-treated filtrated water from 40 m depth with 32ppt salinity. At trial start 100 random fish were weighed and measured to determine the ranges of fish sizes that would be accepted (70% of the initial population) or rejected (lowest and highest 15%) during distribution in the experimental tanks. Each experimental diet was randomly assigned to 3 tanks and fish were fed 15–20% above saturation using automatic belt feeders, distributed in two meals per day for a period of 12 weeks. Uneaten feed was collected and weighed daily for the estimation of total daily feed intake of the experimental fish populations. Water oxygen levels were maintained above 80% saturation and water circulation speed was set at 0.8 L body length^−1^. Mean temperature during the trial was 9.3°C.

At trial end fish in each tank were anaesthetised, individually weighed, and stripped for faeces. Faeces samples were set at −25°C and kept frozen before being freeze dried and analysed for inert marker content (yttrium), proximate composition (crude protein and fat) and gross energy content. Skin (1 × 1 cm right below dorsal fin), Norwegian quality cut (NQC) filet, and blood serum samples were collected from 5 fish per tank. Livers were individually weighed. Blood was withdrawn from the caudal artery using a heparinised vacutainer and centrifuged at 3,000 rpm for 10 min to separate the serum, which was collected and stored at −20°C until analysis. Levels of cortisol were analysed with ELISA using a commercially available kit (Demeditic Diagnostics GmbH, Kiel, Germany), while the rest of the serum analyses were performed by the Pentra Clinical Chemistry Analyzer (Pentra C400, HORIBA ABX SAS, Montpellier, France). Skin samples were analysed for essential trace minerals (Cu, Fe, Mn, Zn and Se), total amino acids and evaluated histologically. NQC samples were analysed for protein, lipids, fatty acid profile and total amino acids.

The feeding experiment followed the Norwegian animal welfare act guidelines, in accordance with the Animal Welfare Act of 20 December 1974, amended 19th of June 2009. The trial facilities were granted permission by the Norwegian Food Safety Authority to run the experiments. The decision was made on the basis of Regulations 18th of June 2015 on the use of animals in experiments, §§ 6, 7, 9, 10 and 11.

### Chemical and Pellet Structure Analyses

The experimental diets, and where relevant fish tissues and faeces, were analysed for protein (Kjeldahl method N x 6.25; ISO 5983–1997), moisture (ISO 6496–1999), ash (ISO 5984–2002) and lipid (Bligh and Dyer, 1959) followed by determination of the oxidation state of the oil by the analysis of peroxide number (AOCS Cd 8b-90) and anisidine number (AOCS cd 18–90). Dietary gross energy was determined in a Parr adiabatic bomb calorimeter. For total amino acid profile determination, samples were hydrolysed in 6 M HCl for 22 h at 110°C and analysed by HPLC using a fluorescence technique for detection (Cohen and Michaud, 1993). Free amino acids, taurine (Tau) and anserine were analysed as described in Bidlingmeyer et al. (1987). The water-soluble fraction of the marine protein meals and the diets was extracted with boiling water, the extract was then filtered using Whatman black ribbon filter paper, and the crude protein content in the water-phase was determined by the Kjeldahl method. Total starch was measured using a modified glucoamylase method described by Chiang and Johnson (1977) and Samuelsen and Oterhals (2016). Total phosphorous (P) was determined by a spectrophotometric method (ISO 6491–1998). Undesirable compounds such as aldrin, dioxins, PCBs, DDT, DDE, TDE, PAH 4 and heavy metals (Hg, Pb, Cd and As) and vitamins in raw materials as well as trace minerals and in raw materials and tissues were analysed by an external laboratory (Eurofins, Hamburg, Germany). The analyses performed externally (Cu, Se, Mn, Zn, Fe, vitamins, and undesirable compounds) were performed in single samples, whereas the remaining analyses were performed in duplicate samples. If differences between parallels exceeded standardised values, new duplicate analyses were carried out according to accredited procedures. More in detail (as provided by Eurofins): For the test trace mineral analyses the sample preparation was realised according to §64 LFGB L.00.00–19/1, CON-PV 00001 (2019–03) with microwave digestion. Copper (Cu), iron (Fe), zinc (Zn) and manganese (Mn) were analysed using ICP-OES according to EN ISO 11885 (modified). Selenium (Se) was analysed using ICP-MS according to an analogue method to §64 LFGB L 00.00–19/3. Retinol (vitamin A) was analysed according to EN 12823–1 2014, alpha tocopherol (vitamin E) was analysed according to EN 12822:2014 and DJCPH L-ascorbyl-2-phosphate (stay-C form of vitamin C added in the diets) was analysed by LC-DAD.

Pellet water stability index (WSI) was used to determine pellet technical quality and was determined by a slightly modified method described by Baeverfjord et al. (2006). Triplicate samples of each diet (20 g each) were added in custom made steel-mesh container placed inside 1,000 ml glass beakers filled with 500 ml distilled water. The beakers were incubated in a thermostat-controlled water bath at 23°C and shaken (160°min^−1^) for 120 min, and the remaining amount of dry matter (DM) was determined.

### Sampling and Calculations

At the end of the trial all fish from each tank, except for 10, of which 5 fish were used for further biological studies and 5 more kept in store, were stripped and their faeces separated from urine and collected in 1 pre-weighed box per tank. Following sampling of each tank the collected faeces were frozen immediately at −20°C prior to further freeze drying and analysis. Apparent digestibility coefficient (ADC) of nutrients and energy in the test diets was calculated from the following formula: ADC = 100 – 100 × [Yd/Yf] × [Nf/Nd] where d is diet, f is faeces, Y yttrium content and N nutrient content.

Fish growth rate, survival, feed intake rates and feed efficiency (TGC: thermal growth coefficient, FCR: feed conversion ratio) and fish biometrics (D%: dress out percentage, HSI: hepatosomatic index, CF: condition factor) were measured. Feed intake was expressed as the total feed consumed per tank, mean feed intake per fish, or mean daily feed consumption per fish expressed as% of its body weight. Feed conversion ratio is feed consumed/biomass increase. Thermal growth coefficient is TGC = (w_final_^1/3^- w_start_^1/3^) x 1,000/degree-days where w is mean fish body weight (Cho, 1992). Condition factor is CF = fish weight (g) x fish fork length^−3^ x 100. Dress out percentage is D% = gutted fish weight/whole fish weight x 100. Hepatosomatic index (HSI) is the% of liver weight/whole fish weight.

Tissue samples were stored in 10% formalin pots (CellStore™ 20 ml Pots, CellPath). Embedding, sectioning, and staining of the tissue samples were done at the Norwegian Veterinary Institute in Harstad, Norway. In brief, the tissue sections were hydrated in water and stained with 1% Alcian blue (Alfa Aesar), 3% acetic acid for 15°min, transferred to 1% periodic acid (VWR) for 10°min, followed by Schiffs (Sigma-Aldrich^®^) reagent for 15°min, 30 s in heamatoxylin (VWR) before dehydration and mounting. The stained tissue sections were scanned with a Hamamatsu slide scanner (Hamamatsu) and uploaded to the Aiforia^®^ platform and analysed according to Sveen et al. (2021).

### Statistics

Biological and analytical data were subjected to one way analysis of variance (ANOVA) tests using IBM SPSS statistics 27 to detect dietary effects. When differences among treatments were identified, means were ranked using the Tukey post hoc test. Equality of error variances was tested with Levene’s test. Effects were considered at a significance level of *p* < 0.05, and tendencies are discussed at *p* < 0.1.

## Results and Discussion

### Raw Material Chemical Characterisation

The analysed fi FM is superior in crude protein content as compared to the rest of the analysed ingredients used in the present trial, followed by black soldier fly larvae meal, *P. tricornutum* biomass, tunicate meal, and *S. limacinum* biomass (Supplementary Table S1). On the other hand, *S. limacinum* meal is a lipid rich source, followed by *P. tricornutum*, FM, black soldier fly meal and tunicate meal (Supplementary Table S6). The FM used in this trial was a rich source of water-soluble peptides, of known growth promoting nutritional value in Atlantic salmon fed low FM diets (Kousoulaki et al., 2009), richer than *P. tricornutum* and black soldier fly larvae meal with lowest values analysed in *S. limacinum* biomass (Supplementary Table S5). Nevertheless, expressed in % of total protein the two microalgal biomasses have equally high levels of water-soluble protein (approx. 40% of total protein) whereas FM and black soldier fly larvae meal contain approx. 23–25% water soluble protein of total protein.

The used test meals had great differences in terms of amino acid composition compared to FM. The FM used in this trial had similar amino acid profile as other conventional FMs, and nearly or more than double the amounts of lysine (Lys) and methionine (Met), respectively, as % in total protein compared to the test ingredients. The black soldier fly larvae meal was also low in cysteine and cystine (Cys), unlike the three other materials which were richer or as rich in both Cys and tryptophane (Trp) as compared to FM. Except tunicate meal, the test ingredients were also lower in arginine (Arg) as compared to FM. The black soldier fly larvae meal contained the highest histidine (His) level, similar to that in FM, and had also similar levels of isoleucine (Ile) and valine (Val) as compared to FM (Supplementary Table S2). The non-dispensable amino acid His is important for osmoregulation (Bjerkås and Sveier, 2004) and prevention of cataract (Breck et al., 2003; Bjerkås and Sveier, 2004) in Atlantic salmon and thus considered in formulating transfer (from fresh to salt water) diets. A considerable part of His in black soldier fly larvae meal was in free form, unlike the remaining test ingredients, where His was either exclusively or mostly in protein bound form. The experimental diets were balanced based on the analysed raw material compositions to be equal in His levels. However, there were analysed lower levels of His in the BSFM diet which may be due to for instance loss of free His due to Maillard reactions during the extrusion process involving the black soldier fly larvae meal. Free His was found to be the most effective free amino acid in breaking down plant polysaccharide chains and reactive with the generated sugars, as it exerted buffering effect eliminating the inhibiting effects of certain compounds (organic acids) on Maillard reaction (Liu et al., 2019). Accordingly, the ADC of His was lowest in BSFM as compared to FMFO, TM, PM, and HM (Table 6). Else, the diets were well balanced in protein bound amino acids, even though we only balanced Lys, Met, Thr, and His in the formulations adding crystalline amino acids. Fishmeal was the raw material with the highest % of non-dispensable amino acids of total protein, followed by tunicate meal, black soldier fly larvae meal and *P. tricornutum* biomass with between them equal levels, and last *S. limacinum* biomass*.*

**TABLE 6:** Experimental diet apparent digestibility coefficient (ADC) of nutrients in Atlantic salmon. Values are in%.

Nutrient	FMFO	TM	BSFM	PM	HM	0FM0FO	*p* value^a^
Protein	91.7 ^d^ ± 0.1	85.3^ab^ ± 1.4	86.6 ^bc^ ±1.9	90.4 ^cd^ ± 0.6	90.5 ^cd^ ± 0.1	81.7^a^ ±3.2	0.000
Fat	95.4^c^ ±0.5	93.3 ^bc^ ±1.2	93.9 ^bc^ ±0.8	93.4 ^bc^ ±0.7	88.4^a^ ±0.9	90.7^ab^ ± 2.8	0.001
Asp	84.8^b^ ± 0.4	79.5^ab^ ± 1.9	78.8^ab^ ± 4.3	84.6^b^ ± 1.1	84^b^ ± 0.7	75.1^a^ ±4	0.002
Glu	97.5^b^ ± 0.2	95.6^b^ ± 0.4	95.9^b^ ± 0.9	96.8^b^ ± 0.4	96.6^b^ ± 0.3	92.8^a^ ±1.3	0.000
Ser	93.5^c^ ±0.6	87.5^ab^ ± 2	89.3 ^bc^ ±2.7	91.3 ^bc^ ±1.2	92.2 ^bc^ ±0.6	84^a^ ±3	0.001
Gly	90.8 ^d^ ± 0.7	84.2 ^bc^ ±1.9	82.6^b^ ± 3.5	88.9 ^cd^ ± 1	88.9 ^cd^ ± 0.8	77.2^a^ ±3.8	0.000
His	96.8^c^ ±0.8	91.7^b^ ± 1	86.3^a^ ±3.2	95.4 ^bc^ ±0.7	95.3 ^bc^ ±0.8	85.1^a^ ±2.7	0.000
Arg	96.2^b^ ± 0.6	90.7^a^ ±1.7	94.4^b^ ± 1.5	95.8^b^ ± 0.2	94.9^b^ ± 0.5	89.6^a^ ±1.8	0.000
Thr	94.3^c^ ±0.3	86.2^b^ ± 1.9	90.3 ^bc^ ±2.01	92.2^c^ ±1	92.5^c^ ±0.7	81.4^a^ ±3.3	0.000
Ala	94.1^c^ ±0.2	88.6^ab^ ± 1.8	90.7 ^bc^ ±1.7	92.8^c^ ±0.7	93.4^c^ ±0.3	86.9^a^ ±2	0.000
Pro	96.3^b^ ± 0.5	93.9^ab^ ± 1.1	94.1^ab^ ± 1.2	95.8^b^ ± 0.5	95.3^b^ ± 0.3	92.1^a^ ±1.3	0.001
Tyr	93.1^c^ ±1	88.4^ab^ ± 0.9	90.7 ^bc^ ±1.9	90.4 ^bc^ ±0.1	91.7 ^bc^ ±0.6	86.1^a^ ±2.3	0.001
Val	93.6^c^ ±0.1	88.9^b^ ± 0.8	89.2^b^ ± 1.7	92 ^bc^ ±0.7	92.5 ^bc^ ±0.6	84.2^a^ ±2.9	0.000
Met	94^b^ ± 0.3	90.2^b^ ± 0.9	93.8^b^ ± 0.5	93.2^b^ ± 0.6	93.3^b^ ± 0.7	89.1^a^ ±1.8	0.000
Ile	94.2 ^d^ ± 0.2	89.5^b^ ± 0.9	90.3 ^bc^ ±1.7	93.1 ^cd^ ± 0.6	93.4 ^cd^ ± 0.6	86.3^a^ ±2.4	0.000
Leu	95.2^c^ ±0	91.5^b^ ± 0.9	92.73 ^bc^ ±1.1	94.3^c^ ±0.3	94.4^c^ ±0.4	88.4^a^ ±1.9	0.000
Phe	93.1^c^ ±0.1	89.2^ab^ ± 0.9	91.2 ^bc^ ±1.4	92.7^c^ ±0.4	92.8^c^ ±0.4	86.6^a^ ±2.3	0.000
Lys	95.3^c^ ±0.3	92.1^b^ ± 0.8	92.5 ^bc^ ±1.8	94 ^bc^ ±0.6	94.1 ^bc^ ±0.4	88.2^a^ ±1.9	0.000
14:0	97.5^c^ ±0.7	95.2^b^ ± 1	96.3^b^ ± 0.8	96.1^b^ ± 0.8	69.6^a^ ±3.1	78.8^b^ ± 7.3	0.000
16:0	96^c^ ±0.8	93.9^c^ ±1	94.8^c^±0.7	94.6^c^ ±0.8	66.5^a^ ±3.2	75.8^b^ ± 8.2	0.000
18:0	95.7^b^ ± 0.8	92.9^b^ ± 1	93.6^b^ ± 0.6	94.4^b^ ± 1	88^a^ ±1.1	91.7^b^ ± 3	0.001
20:0	97.5^b^ ± 1	94.2^b^ ± 1.1	93.9^b^ ± 0.8	97.2^b^ ± 2.4	88.4^a^ ±0.9	93.8^b^ ± 1.9	0.000
22:0	94 ± 2.5	89.2 ± 3.3	90 ± 2.8	93.4 ± 0.7	88.4 ± 0.9	71.3 ± 35.6	Ns
**Saturated fatty acids**	**96.4** ^**c**^ **±0.7**	**94.1** ^**c**^ **±1**	**95.1** ^**c**^ **±0.7**	**95** ^**c**^ **±0.8**	**69.6** ^**a**^ **±2.9**	**78 ** ^**b**^ **± 7.5**	**0.000**
16:1 n-7	98.2^b^ ± 0.3	97.8^b^ ± 0.5	97.4^b^ ± 0.7	96.8^b^ ± 0.4	94.6^a^ ±1.1	97.7^b^ ± 0.7	0.000
18:1 (n-9)±(n-7)±(n-5)	98.1^b^ ± 0.3	97.7^ab^ ± 0.5	96.9^a^ ±0.6	97.4^ab^ ± 0.4	96.8^a^ ±0.2	99.1^c^ ±0.2	0.000
20:1 (n-9)±(n-7)	97.6 ^cd^ ± 0.3	96.4 ^bc^ ±0.7	96.2^b^ ± 0.7	96.5 ^bc^ ±0.5	94.9^a^ ±0.4	97.9 ^d^ ± 0.3	0.000
22:1 (n-11)±(n-9)±(n-7)	97.2^c^ ±0.3	95.8 ^bc^ ±0.7	95.7 ^bc^ ±0.6	95.8 ^bc^ ±0.8	92.3^a^ ±0.5	94^ab^ ± 2.5	0.003
24:1 n-9	95.1^b^ ± 0.4	92.9^b^ ± 1.6	93.3^b^ ± 1.7	93.4^b^ ± 1.7	88.4^a^ ±0.9		0.001
**Monounsaturated fatty acids**	**97.8** ^**b**^ **± 0.3**	**97.1** ^**ab**^ **±0.5**	**96.5** ^**a**^ **±0.6**	**96.8** ^**ab**^ **±0.5**	**96.5** ^**a**^ **±0.2**	**99** ^**c**^ **±0.2**	**0.000**
18:2 n-6	97.4^b^ ± 0.2	97.4^b^ ± 0.4	96.9^ab^ ± 0.3	96.9^ab^ ± 0.3	96.5^a^ ±0.3	98.9^c^ ±0.4	0.000
**PUFA (n-6) fatty acids**	**97.5** ^**b**^ **± 0.2**	**97.5** ^**b**^ **± 0.4**	**96.9** ^**ab**^ **±0.3**	**97** ^**ab**^ **±0.3**	**96.6** ^**a**^ **±0.3**	**98.9** ^**c**^ **±0.3**	**0.000**
18:3 n-3	99^c^ ±0	99.2^c^ ±0.3	98.5^b^ ± 0.3	98.4^ab^ ± 0.3	98^a^ ±0.2	99.7 ^d^ ± 0.1	0.000
20:5 n-3 (EPA)	98.9 ^bc^ ±0.1	98.7 ^bc^ ±0.3	98.6 ^bc^ ±0.2	98.3^b^ ± 0.2	96.7^a^ ±0.2	99^c^ ±0.3	0.000
22:5 n-3	96.1^b^ ± 0.6	95.3^b^ ± 1.4	96.7^b^ ± 2.9	94.2^b^ ± 2	88.4^a^ ±0.9	94^b^ ± 2.5	0.002
22:6 n-3 (DHA)	97.9^b^ ± 0.2	97.7^b^ ± 0.4	97.7^b^ ± 0.1	97.4^b^ ± 0.3	95.4^a^ ±0.5	98^b^ ± 0.7	0.000
**PUFA (n-3) fatty acids**	**98.6** ^**ab**^ **±0.1**	**98.7** ^**ab**^ **±0.4**	**98.3** ^**ab**^ **±0.3**	**98 ** ^**b**^ **± 0.3**	**97** ^**a**^ **±0.3**	**99** ^**c**^ **±0.3**	**0.000**
PUFA fatty acids	98.2^b^ ± 0.1	98.2^b^ ± 0.3	97.8^b^ ± 0.3	97.6^b^ ± 0.3	96.8^a^ ±0.2	99^c^ ±0.3	0.000
EPA ± DHA	98.4^b^ ± 0.1	98.1^b^ ± 0.4	98.2^b^ ± 0.2	97.8^b^ ± 0.3	95.5^a^ ±0.5	98.1^b^ ± 0.6	0.000
Identified fatty acids	97.7^c^ ±0.3	96.9^c^ ±0.5	96.6^c^ ±0.5	96.8^c^ ±0.5	91.2^a^ ±0.8	94.2^b^ ± 1.9	0.000
Unidentified fatty acids	98.3^c^ ±0.8	96.6^c^ ±0.4	97.5^c^ ±0.4	97.1^c^ ±0.8	82.6^a^ ±1.6	92.4^b^ ± 2.6	0.000
Cu	19.9 ± 3.1	17.5 ± 18.4	21.2 ± 11.9	15.5 ± 0.9	8.8 ± 3.04	14.3 ± 21.1	ns
Fe	−8.0^a^ ±10.8	12.2^ab^ ± 6.5	15.4^ab^ ± 10.1	−2.3^a^ ±5.7	5.0^ab^ ± 5.7	24.0^b^ ± 15.2	0.015
Zn	3.1 ± 10.4	-4.6 ± 17.1	11.2 ± 14.3	−1.8 ± 1.7	−5.26 ± 10.5	1.6 ± 21.8	ns
Se	50.0 ± 11.8				46.0 ± 11.7	28.2 ± 25.3	ns
Mn	−21.1 ± 24.2	10.1 ± 61.9	6 ± 13.8	1.7 ± 6.6	−20.8 ± 16.4	−12.9 ± 25	ns

a Values in the same line with different small superscript letter are significantly different (*p* < 0.05) following Tukey post hoc test.

*Phaeodactylum tricornutum* biomass was the raw material with the highest % of free amino acids in the water-soluble protein fraction (Supplementary Table S2), and other nitrogenous compounds of low molecular weight (<200 Da), followed by *S. limacinum* biomass, FM, black soldier fly larvae meal, and tunicate meal. Tunicate meal and *S. limacinum* biomass had the highest % of small peptides (200–2000 Da). Black solider fly larvae meal, tunicate meal and FM had the highest levels of medium sized peptides (2000–15,000 Da). Finally, the two analysed FMs and the tunicate meal had the highest levels of larger soluble peptides (>20,000 Da) (Supplementary Table S3).

In terms of free amino acids, FM was richest in free creatinine and Tau and had in total similar total free amino acid levels as those analysed in black soldier fly larvae meal and *S. limacinum* biomass. Tunicate meal had the lowest level of free amino acids among the analysed raw materials. The predominant free amino acids in black soldier fly larvae meal were alanine (Ala), Arg, proline (Pro), and tyrosine (Tyr) (Supplementary Table S2). *Phaeodactylum tricornutum* biomass was the richest source of free amino acids among the analysed raw materials. The most abundant free amino acids in *P. tricornutum* biomass were the dispensable amino acids glutamic acid (Glu; 1.245% in the diet), Pro (0.955% in the diet), Ala (0.85% in the diet), glycine (Gly; 0.485% in the diet), ornithine (Orn; 0.435% in the diet) all but Orn also found overrepresented in krill hydrolysate and correlating with increased feed intake levels (Kousoulaki et al., 2013) and feed searching activity (Hara, 2006; Hara et al., 1993) in salmonids. All free amino acids analysed in *S. limacinum* biomass were found in concentrations between 0.0 and 0.1% (Supplementary Table S2). Fishmeal was richest in Tau and creatinine compared to the rest of the test ingredients, which are water soluble nitrogenous compounds present in the water-soluble fraction of FM (stickwater) and their dietary level was found to correlate with feed intake rates and growth in Atlantic salmon (Aksnes et al., 2006; Kousoulaki et al., 2009).

EPA-DHA levels ranged between 9.5 and 27% of total lipids in the assessed marine dietary ingredients. *S. limacinum* contained the highest levels of sum EPA ± DHA (mainly DHA) in the lipid fraction of the biomass as compared to the other marine sources analysed, followed by FM, *P. tricornutum* biomass, FO and tunicate meal. Other interesting facts regarding the assessed marine raw material lipids are the high level saturated fatty acids in *S. limacinum* biomass (approx. 62% of total lipids), the high levels of monounsaturated fatty acids of FO (48.5% of total lipids) and the high levels of unidentified fatty acids in the tunicate meal lipid extract (76.3% of total Bligh & Dyer (B&D) extract (1959)) which deserves further exploration.

Black soldier fly larvae meal contained approx. 10% lipids, with a rather high levels of unidentified fatty acids too. The main fatty acids present in black soldier fly larvae meal were saturated fatty acids, followed by n-6 PUFA and monounsaturated fatty acids with no EPA or DHA present at any significant amounts (EPA ± DHA = 0.1% of total lipid extract) (Supplementary Table S6).

The lipids analysed in *P. tricornutum* biomass were mostly in free fatty acid form with presence of cholesterol esters. Though absent from higher plant oils, marine microalgae (Vernon et al., 1998; Volkman et al., 1989) as well as the freshwater species *Nannochloropsis limnetica* (Martin-Creuzburg and Merkel, 2016) can produce and store cholesterol, besides other sterols. Sterols are essential nutrients for crustacean zooplankton, but different phytosterols were found to support better growth in *Dafnia magna* than cholesterol (Martin-Creuzburg et al., 2014). Aquafeed cholesterol levels are reducing by substitution of FO with plant oils, as for instance soy and rapeseed oil that are devoid of cholesterol (Norambuena et al., 2013). Cholesterol has vital physiological roles in animals and though it can be endogenously synthesized by fish including Atlantic salmon (Leaver et al., 2008), it has been stipulated that dietary supplementation of cholesterol may provide benefits (Norambuena et al., 2013). Polar lipids in *P. tricornutum* were also analysed at higher levels as compared to *S. limacinum* which was in turn richer in triacylglycerols (Supplementary Table S7). Only 36% of the extracted lipid fraction in *P. tricornutum* was identified in lipid classes as compared to over 55% identified as fatty acids in the same raw material. Almost 100% of FO and *S. limacinum* biomass B&D extract were accounted for in the different identified lipid classes. The analysed FM B&D extract was richest in polar lipids as compared to the other analysed ingredients, with high levels of phosphatidylcholine. *S. limacinum* B&D extract was rich in tripalmitin (over 30% of the lipid extract), which is assumed to be highly indigestible in Atlantic salmon (Kousoulaki et al., 2015).

Aerobic microorganisms were present at highest levels in the black soldier fly larvae meal (410,000 KDE/g), followed by the processed *P. tricornutum* biomass (1,600 KDE/g), *S. limacinum* (460 KDE/g) and FM (230 KDE/g) (Supplementary Table S5). Bacteria are originally present at high levels in fish and seafood (Nickelson and Finne, 1992) and are growing in the cultivation medium together with microalgae or in the insect feed medium but are expected to be drastically reduced by heat treatment or cell wall disruption in the case of microalgae (e.g., Doucha and Lívanský, 2008). The Norwegian Food Authorities guidelines limit the number of aerobic bacteria in feed exported to countries withing the Euro-Asiatic Economic Zone to 500,000 KDE/g, which is higher than that analysed in the test ingredients used in this study. Though no official limits are defined, the presence of high aerobic bacterial count may indicate lower raw material quality and shelf-life due to contamination or insufficient heat treatment. The *P. tricornutum* biomass used in our study was a fresh and mild processed (bead milled and spray dried) biomass with nearly no detectable levels of histamine, putrescin or cadaverine. The *S. limacinum* biomass was heat processed to kill the cells in culture followed by spray drying and was low in microbial load, but it contained the highest analysed levels of putrescine followed by FM and black soldier fly larvae meal. The levels of biogenic amines putrescine, cadaverine and histamine detected in FM and *S. limacinum* biomass were 10 or more times lower than those analysed in FM produced by stale herring but higher than those analysed in FM produced by fresh herring (Opstvedt et al., 2000). Putrescine, cadaverine and histamine do not appear to be a hazard for fish and do not affect Atlantic salmon performance when added in the diet at levels equivalent to those present in FM produced by stale fish, but they provide indication of raw material quality significantly and negatively affecting fish performance (Aksnes and Mundheim, 1997; Opstvedt et al., 2000).

The tested FM was richer in vitamin A compared to the other analysed test meals, and contained significant amounts of vitamin E, which is probably due to the use of tocopherols as antioxidant in the raw material against ethoxyquin that was previously used in conventional FM. In June 2017 the EU commission suspended the organic authorisation of ethoxyquin for all animal species and categories with a transition period to introduce alternative antioxidants until 2020 (EU Regulation 2017/962). The levels of vitamin A in FM (5.47 mg/kg) were approx. 10 times the amount provided in the trial diets by the vitamin mixed used intended to cover the requirement of Atlantic salmon (Table 1). Vitamin A was analysed also in *S. limacinum* biomass (1.23 mg/kg) which was the only vitamin of those analysed present in this raw material. Vitamin D3 was identified only in FM (0.053 mg/kg) at levels equivalent to those added by the dietary vitamin mix (0.0625 mg/kg diet), which is just above the minimum level of the estimated requirement of Atlantic salmon in sea water (0.06–0.09 mg/kg diet) (Antony Jesu Prabhu et al., 2019). The *P. tricornutum* biomass was naturally rich in both tocopherols (vitamin E) (156 mg/kg) and vitamin C (1,050 mg/kg ascorbic acid). The dietary vitamin mix provides approx. 200 ppm vitamin E in the final diet, which is equivalent to that present in the algal biomass and 4 times that present in the test FM. The requirement of fish in vitamin E depends on and can be covered by adequate levels of vitamin C and Se in the diet (El-Sayed and Izquierdo, 2021) and was found to be higher than 60 mg/kg in Hamre et al. (1994). Vitamin C was only analysed in *P. tricornutum* biomass, where it appears to be present in a stable form, unlike in fish where it is known to perish rapidly during processing during fishmeal or compound feed production (Putnam, 1976). The minimum requirement of Atlantic salmon fry in vitamin C at start feeding was estimated to be 10–20 mg/kg dry diet (Sandnes et al., 1992), which is covered in commercial and research feeds by dietary addition of a stable form of ascorbic acid (stay-C: phosphorylated l-ascorbic acid) (Grant et al., 1989) and could alternatively also be covered by 1–2% dietary inclusion of the *P. tricornutum* biomass used in our study or other microalgal biomasses with similar properties. Excessive levels of vitamin C may be present when using more than 2% phototrophic microalgae biomass in the diet such as is the case in the present study. Inconsistent data exist in the literature regarding the safety of high vitamin C levels in fish diets, including suspected negative effects in Atlantic salmon survival and growth by inclusion of approx. 1,580 mg/kg vitamin C combined with 450 mg/kg vitamin E in the diet (Kousoulaki et al., 2021), improved immune responses and disease resistance of Atlantic salmon pre-smolt fed up to 4,000 mg/kg diet vitamin C (Waagbo et al., 1993) or no effect by feeding juvenile eel a diet with 1,137 mg/kg vitamin C (Bae et al., 2012). Regarding K vitamins, K1 was analysed only in tunicate meal and *P. tricornutum* at comparable levels (Supplementary Table S8).

In terms of essential trace minerals, all analysed ingredients were found to be good sources of Fe (Supplementary Table S8). However, tunicate meal was found to contain excessive Fe levels, whereas black soldier fly larvae meal in turn, contained very high Mn levels, as also appears to be the case in Belghit et al. (2018) where diets containing 60% insect meal were analysed to have 160 mg kg^−1^ higher Mn levels as compared to those containing FM and soy protein concentrate instead. In the same study it appears that the insect meal used also contained higher levels of Fe than the replaced ingredients, which was not the case in our study. The maximum allowed levels of Mn in aqua feeds for Atlantic salmon is 100 mg kg^−1^ and that of Fe is 750 mg kg^−1^ (Regulation (EC) No 1831, 2003), which would render the supplementation of these trace minerals in diets containing significant amounts of insect meal and tunicate meal unnecessary. Organic fishmeal and tunicate meals were found to be good sources of Se. Zn was found in higher levels in tunicate meal and black soldier fly larvae meal whereas it was present in significantly lower levels in the FM.

The analysed test raw materials contained generally low levels of undesirable compounds and within the limits defined by the Directive 2002/32/EC and the amended Regulation (EC) No 1881/2006 (Supplementary Table S9). Organic fishmeal had higher levels of undesirable compounds as for instance dioxins and non-dioxin like PCBs and heavy metals (Pb, As, Hg, Cd) as compared to the other test raw materials analysed. This does not come as a surprise, as *P. tricornutum*, *S. limacinum* and insects are grown in controlled environment and can be produced on cleaner nutrient sources than wild fish foraging in proximity of industrial areas (Nøstbakken et al., 2018). The level of heavy metals and persistent organic pollutants (POPs) in organisms growing wild vary largely by genera. The mechanisms of heavy metal accumulation also differ from that of POP accumulation (Landis et al., 1993) but there are a few ecotoxicological generalisations that can be applied to tunicates as a feed-ingredient. Tunicates are grown in the sea and are, generally, considered to eat mostly phytoplankton and are as such low in the trophic food chain. Short-lived organisms accumulate less pollutants than the long-lived organisms. Tunicates are harvested as feed ingredient when they are half-a-year to maximum 1 year old (Grzimek 1972). The production period for tunicates in East Atlantic waters at ∼58°N is 1 year. The species used in this study (*Ciona intestinalis*) spawn in mid-May to mid-June (Dybern 1965) and are ready to be harvested in September. Harvest starts when the animals reach ∼5 cm in length. After spawning, one-year-old individuals die (Fredrik Norén unpublished data). Forage fish of higher trophic level have longer life span, e.g., up to 6 years for Pacific sandfish caught in southeaster Alaska (Thedinga et al., 2006) and over 4 years that is the reproduction age of Atlantic herring (https://www.fisheries.noaa.gov/species/atlantic-herring) before capture, thus resulting in lower accumulation levels of contaminants in their tissues.

The test FM, the *P. tricornutum* and *S. limacinum* biomass and the black soldier fly larvae meal used were also analysed for their content in nucleotides which can be used as indication of muscle degradation in fish (ATP and metabolites) (Supplementary Table S4). The test FM contained larger amounts of hypoxanthine, IMP, and inosine, due to the potential larger relative amounts of intestines with higher enzymatic activity prior to heat treatment in FM production as compared to the remaining ingredients. Black soldier fly larvae meal contained higher levels AMP (Adenosine monophosphate) compared to the other ingredients and similar levels ADP (Adenosine diphosphate) as *P. tricornutum* biomass. Nucleotides can have bioactive function in diets, acting as growth promoters or appetite stimulators (Dias et al., 1997; Yilmaz, 2005; Li and Gatlin, 2006). Nevertheless, Ikeda et al. (1991) found that, in jack mackerel, only some nucleotides exert chemoattractant effect, including IMP present in higher amounts in FM as compared to the other test ingredients in our study, but not ADP or AMP that were present in higher amounts in black soldier fly larvae meal as compared to FM. Accordingly, Rumsey et al. (1992) found negative effects in feed intake and growth in rainbow trout (*Oncorhynchus mykiss*) given a diet supplemented with free adenine (purine), whereas no such negative effect was seen by respective dietary increases in whole yeast extract or other purines such as guanine and xanthine, that exerted positive feeding response, and hypoxanthine that had no effect. Nevertheless, differences in dietary crude protein levels among the different test diets in the above-mentioned study, render, according to the authors, the conclusions on the direct negative effect of adenine ambiguous. Uauy et al. (1990) found growth increase and differentiation of the developing gastrointestinal tract in rats added AMP in the diet, and Kousoulaki et al. (2013) found no effect in growth or feed intake adding 0.18% AMP in the diet of Atlantic salmon. In any case, raw material freshness and the quality and amount of non-protein nitrogen fraction in novel single cell raw materials should be critically considered when incorporated at high levels in the diets of fish, and more specific studies are needed to reveal potential negative effects.

### Pellet Technical Quality of Experimental Diets

**FIGURE 1:**
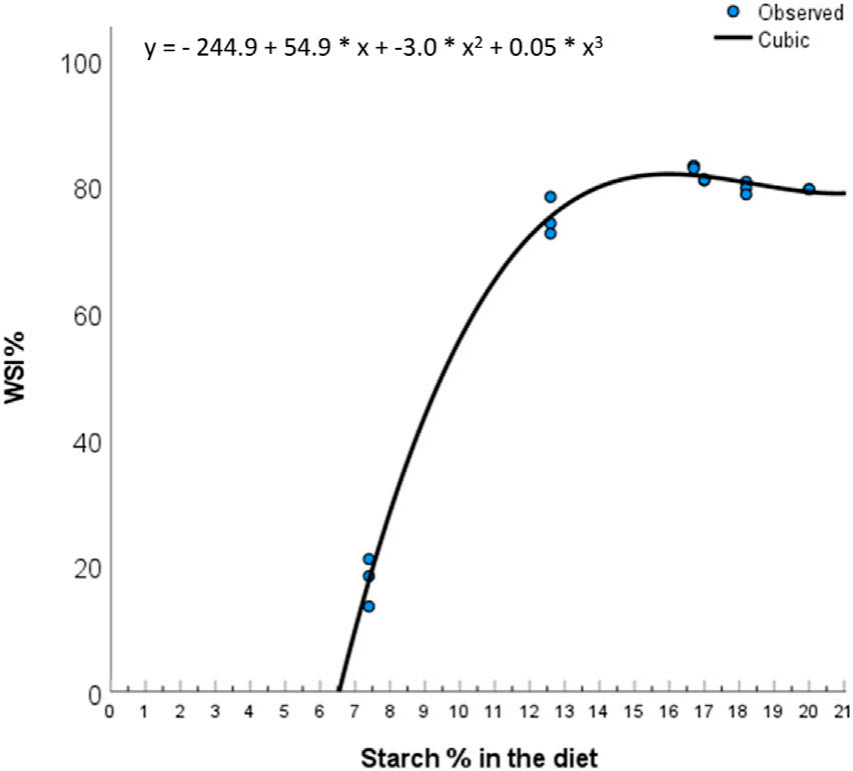
Relation between dietary starch levels and dry matter water stability. Best fit of data was achieved by a cubic model (Total degrees of freedom = 16; R = 0.997; *R* ^2^ = 0.994, adjusted *R* ^2^ = 0.993, standard error of the estimate = 2.112; ANOVA *p* < 0.001).

Highest water stability index (WSI) (approx. 20%) was analysed in the BSFL diet, followed by PM and HM diets, then the FMFO diet, the TM diet and last, the 0FM0FO diet. The FM0FM0 diet had too low WSI to estimate the amounts of uneaten feed in a reliable way. Thus, uneaten feed collection was not performed in this treatment. Pellet quality and technical characteristics depend on among other the chemical properties of the ingredients used (Sørensen 2012). The low water stability of extruded pellets, as is the case in our study, relates well with the amounts of starch present in the diet (Figure 1), which varied due to balancing the diets, using ingredients with very different proximate composition, for adequate and equal protein and lipid levels for Atlantic salmon smolt to the expense of mainly carbohydrates. As a rule of thumb in feed production in Aquafeed Technology Center (ATC) (Nofima, Bergen, Norway) (Personal communication with Dr. Odd Helge Romarheim) we consider 10% starch to be enough to produce water stable pellets. Based on Figure 1, we see that the increases in WSI are low for dietary starch levels above 12.5%. The TM diet contained lower levels of starch as compared to BSFM, FMFO, PM and HM diets, and had lower WSI. Dietary starch levels may have not been the only reason for the observed effect, as in Samuelsen et al. (2022) high levels of tunicate meal inclusion tended to result in lower pellet water stability irrespective of dietary starch levels. On the other hand, the FMFO diet contained the highest levels of starch but did not have the highest WSI. Fishmeal physicochemical and rheological properties are known to greatly affect pellet quality characteristics (Samuelsen et al., 2013; Samuelsen et al., 2014; Samuelsen and Oterhals, 2016). The relationship between technical and nutritional quality of extruded fish feed pellets is a little studied research area that deserves more attention.

### Fish Performance and Blood Metabolites

We saw significantly higher growth rate in the FMFO treatment as compared to the rest. Fish TGC was similar and high (approx. 4) among the single test ingredient experimental groups and higher as compared to the 0FM0FO treatment. Growth mostly correlated with feed intake, thus FCR was similar among treatments, except for the TM treatment where FCR was higher than in the other treatments (but also in general rather low) which may be due to the higher inert ash levels present in the TM diet. TGC correlated significantly and positively with serum K, aspartate aminotransferase (ASAT), alanine aminotransferase (ALAT) and creatitine cinase CK. Thought higher blood levels of these liver and skeletal muscle metabolites are considered in context of physiological stress as for instance during exposure to pathogens (e.g., Rojas et al., 2018) or contaminants (e.g., Yancheva et al., 2014), it appears that they may also correlate with higher cellular and metabolic turnover among equally healthy fish. ASAT and ALAT are enzymes involved in amino acid metabolism, and similarly to our study, Gaye-Siessegger et al. (2007) measured higher liver ASAT values in Nile Tilapia of higher growth rates.

Belghit et al. (2018) used 60% black soldier fly larvae meal replacing 29% of FM and 24.5% soy protein concentrate in three different diets for Atlantic salmon and saw no significant reduction in fish growth performance, though the ADC of protein and nearly all amino acids considered in the insect meal-based diets were significantly lower than that of the respective FM-based diets. This may be because the insect meal-based diets in the study of Belghit et al. (2018) contained significantly higher levels of lipids and gross energy compared to the high FM diets which may have contributed to increased growth rate, and also may have contributed to the increase in relative liver weight that the authors report. Similar inclusion levels of *S. limacinum* (Kousoulaki et al., 2020b) and *Ciona intestinalis* (Kousoulaki et al., 2020a) have yielded similar or better (in the case of *S. limacinum*) performance results in Atlantic salmon as compared to FM/FO based diets, but then the experimental diets and FM and FO raw material qualities were different.

The superior performance of the FMFO treatment is in agreement with the ADC results of this study, showing that FM had higher ADC of protein as compared to the alternative protein sources (black soldier fly larvae and tunicate meals) and FO had higher ADC of total lipids and most fatty acids as compared to the lipids that the microalgal ingredients contributed to the diet with. Dietary nutrient ADC was significantly affected by dietary treatment for most nutrients analysed except Cu, Zn, Se, Mn and gross energy and 22:0. The diets with alternative protein sources (black soldier fly larvae meal and tunicate meal) had lower ADC of protein (86.6 and 85.3%, respectively) than the FMFO (91.7%) diet. Their combination and complete removal of the higher digestible FM reduced ADC of protein further in the 0FM0FO treatment (81.7%). Similar picture was shown for ADC of most amino acids, though their dietary levels were balanced, which may indicate suboptimal processing of these new raw materials still in development (e.g., excess heating). Ramos-Elorduy et al. (1981) reported protein digestibility in different insect meal between 45 and 66.9%. Nogales-Mérida et al. (2019) reviewed the ADC of nutrients of different insect meals in fish and reported higher ADC of crude protein in black soldier fly larvae meal (81.1–97.0%) as compared to than those obtained in *Tenebrio molitor* larvae meal (79.2–92.0%) or *Zophobas morio* larvae meal (50.5%). Belghit et al. (2018) reported lower amino acid ADC and a 3% decrease in ADC of protein in Atlantic salmon fed 60% black soldier fly larvae meal in the diet against a control diet with 35% FM inclusion. Moreover, in Belghit et al. (2018) ADC of protein in the insect rich diet was higher (approx. 93–94%) than in our study (87%), probably due to fish size difference (50–143 g fish in Belghit et al. (2018) against 140–450 g in the current study) but also differences in raw material quality and dietary formulation. According to Jonas-Levi and Martinez (2017), the observed differences in apparent crude protein digestibility of insect meals in the literature are due to overestimation of dietary protein level due to the presence of chitin. However, this explanation does not cover the observed differences in individual amino acids seen e.g., in both Belghit et al. (2018) and our current study. Though arthropods, including insects have been part of fish’s natural pray since prehistoric times (Maisey 1994), lower insect meal protein digestibility is probably also due to the presence of high levels of the crude fibre chitin. Chitin is part of the insects’ exoskeleton, previously shown to limit rainbow trout growth performance as it was little digested despite the presence of relatively high levels of chitinase activity in the stomachs and of chitobiase in the intestines of the fish (Lindsay et al., 1984; Matsumiya et al., 2006). ADC of protein, some non-dispensable amino acids (Lys, Ile, Val and His) and lipids in the *P. tricornutum* treatment reduced as compared to the FMFO diet, but not statistically significantly.

In the present study, we observed lower fish growth performance when replacing FM with phototrophic microalga *P. tricornutum* cell wall disrupted biomass, contrary to Kiron et al. (2016) where only FCR but not growth was affected by 10 and 20% dietary inclusion of defatted *Desmodesmus* sp. biomass. In our study lower growth was related to lower feed intake rates in the PM treatment. The FCR in the PM treatment was numerically lowest among that of the other treatments with single novel ingredient dietary inclusion, though not statistically significantly, and equal to that of the FMFO. ADC of lipids was lowest in the HM diet, as reported in short term trials before (Kousoulaki et al., 2015) due to the high levels of tripalmitin in *S. limacinum* oil (Bogevik et al., 2018). The HM diet had also slightly lower ADC of MUFA, n-6PUFA, n-3PUFA and EPA ± DHA as compared to the FMFO and other test diets, except the 0FM0FO which had surprisingly the highest levels among all the groups (Table 6). In previous studies where *S. limacinum* was used in test diets to replace FO, the control diets included higher levels of palm oil and lower levels of linen oil to balance all experimental diets for total saturated fatty acids and n-3/n-6 dietary profiles. This resulted, in short term to lower saturated fatty acid ADC (Miller et al., 2007; Kousoulaki et al., 2015; Kousoulaki et al., 2016), but in longer term, to higher lipid and protein ADC in the *S. limacinum* groups (Kousoulaki et al., 2020b). The reasoning behind this design was to identify physiological dietary effects in Atlantic salmon from decreasing EPA/DHA ratio in the dietary lipids, which is the consequence of replacement of FO by *S. limacinum*, without the confounding effects of other lipid group imbalances. In our present trial, we aimed to reveal novel raw material limitations and balanced essential nutrients such as for instance Lys, Met and EPA + DHA, as well as for nutrients and dietary characteristics with known effects on fish health, such as the ratio of proinflammatory n-6 PUFA to anti-inflammatory PUFA. Nevertheless, despite the differences in ADC, fish in the *S. limacinum* treatment performed equally to those in BSFL and TM, and better than those in 0FM0FO treatments, showing that ADC is not always a good indicator for predicting growth performance or FCR and that differences in dietary saturated fatty acid amount and quality can affect protein and energy metabolism in a not always predictable manner.

Lipid ADC was significantly higher in PM as compared to the HM, with higher ADC of all fatty acids analysed (SFA, MUFA, n-3 and n-6 PUFAs), possibly due to the relatively higher FO level in the PM diet combined with the higher ADC of *P. tricornutum* lipids as compared to those in *S. limacinum* biomass. The *P. tricornutum* biomass used in PM was cell wall disrupted according to an optimised process using bead milling (Kokkali et al. manuscript) and spray dried before feed production. Spray drying is a mild drying process safeguarding raw material quality properties (Ameri and Maa, 2006). Still, using *P. tricornutum* biomass in the diet of Atlantic salmon in our study, as when using other autotrophically produced microalgal biomasses, yielded lower fish performance as compared to FM and FO, which may be due to different factors, such as for instance partial cell wall integrity though the biomass was both pre-disrupted and the diet produced by extrusion which is also known to improve microalgae nutrient availability (Wang et al., 2018; Gong et al., 2020), digestion disturbance (Jafri, 1998; Refstie et al., 1999) and gut microbial imbalance by microalgal carbohydrates (Desai et al., 2012). Even the presence of too high levels of vitamins and other antioxidant compounds such as phenolics present in phototrophic microalgae (Khan et al., 2018) may disrupt normal digestive physiology in fish, which can be approached by biorefinery processes (Bongiorno et al., 2022) separating and using the different biomass fractions in appropriate applications and dietary levels.

Apparent digestibility of minerals was, as often seen, difficult to relate to performance. Statistically significant differences were only seen in ADC of Fe, with higher values in the best and lower values (negative) in the lowest performing groups.

### Fish Biometrics and Tissue Composition

The fish biometric indices showed little variation and small significant differences among the treatments (Table 7). Fish in 0FM0FO treatment were the smallest and also had the lowest CF and D%. A characteristic difference among treatments was the higher relative liver size (HSI) in BSFL treatment as compared to the FMFO which agreed also with the analysed higher lipid levels in the fillets (NQC) of fish in this treatment. This result may be related to the lower levels of the non-dispensable amino acid His in the BSFL diet and the generally lower ADC of non-dispensable amino acids in this diet as compared to FM, which may in turn have induced higher amino acid catabolism for use as energy source resulting in higher lipid deposition in body tissues. Nevertheless, according to this theory we should have observed the same effects in the TM treatment which was not the case. Belghit et al. (2018) and Lock et al. (2016) also observed increased HSI in Atlantic salmon fed diets with high inclusion levels of black soldier fly larvae meal and attributed the effect to suboptimal lipid metabolism due to lack of Tau in the low FM diets. This hypothesis does not appear to be true in the case of our study. The trial diets of the current study contained equal levels Met. The 0FM0FO and BSFL diets contained the same dietary level of black soldier fly larvae meal (20%) and as such the lowest and equally among them (calculated) Tau levels among the experimental diets. However, only in the BSFL treatment fish had significantly higher HSI values as compared to the remaining treatments, but not in the 0FM0FO, which had equal HSI values as e.g., the FMFO treatment containing the highest dietary Tau level.

Drawing conclusions on simple correlations entails significant risk of ending up with false conclusions. For instance, HSI values in this study correlate significantly (two tailed Pearson’s correlation; *p* < 0.05) and negatively with dietary soluble P, 18:1 and 20:0 fatty acids, dietary serine, glycine, His and isoleucine, and also with the response parameter fillet protein, and no other parameter. This occurs obviously because the BSFL treatment has the highest HSI and also the lowest levels, through only marginally of the above listed dietary factors, that are equal among the control and remaining test diets. On the other hand, the negative correlation between fillet protein levels and fish HSI is more interesting. Fillet protein levels also correlate (two tailed Pearson’s correlation; *p* < 0.05) significantly with dietary His, which was low in the BSFL diet, despite the originally analysed high levels in the raw material, dietary isoleucine and serine, differing only marginally in the diets. Interestingly fillet protein also correlates positively with fillet Zn levels and negatively with ADC of dietary Zn. This fact, combined with the much higher Mn levels in BSFL diet, may indicate that Zn uptake was hindered by competition with Mn, possibly disturbing both lipid and protein metabolism, where both Zn and Mn are involved as cofactors in key enzymatic processes (Greenwood and Earnshaw, 1997). Prabhakaran et al. (2008) propose that in humans, the mechanism behind manganese toxicity induced by high levels of Mn inhalation is dysregulation of physiological processes involving oxidative stress, mitochondrial dysfunction, glutamate-mediated excitotoxicity, and aggregation of proteins. It was previously reported that organic minerals, including Zn improve dietary acid digestibility, and reduce fillet gaping in Atlantic salmon (Kousoulaki et al., 2016). Eder and Kirchgessner (1994a;b) in their work in mammalian model animals show the role of dietary Zn in the elongation of alpha linolenic acid to EPA and DHA and that Zn deficiency is demonstrated in both brain and liver lipid profile of the animals. In our study. This agrees with the observations in our study where Atlantic salmon in the BSFL treatment, had the lowest EPA + DHA levels in the fillet, though the respective diet did not contain the lowest amounts of EPA and DHA. It is thus a plausible assumption that some of the main effects we have seen using black soldier fly larvae meal are due to its very high level of Mn and there by inhibition of Zn uptake and related functions. If this is true, insect meal producers should aim to reduce the level of this trace element in their product.

The rest of the treatment differences in NQC fatty acid profile can be explained by the differences in the fatty acid composition of the dietary lipids (Table 2). Nevertheless, at trial end, filets lipids in all treatments contained approx. 16% saturated fatty acids (SFA), whereas SFA spanned from 13.1 to 18.3% of total dietary fatty acids. The monounsaturated fatty acid (MUFA) levels in Atlantic salmon NQC were lower than in the respective diets when those contained approx. 45% of total lipids MUFA and higher than in the respective diets when dietary MUFA levels were below 40%. Filet and dietary n-6 PUFA fatty acids were the same. Last, filet n-3 PUFA were slightly higher than in the respective diets, except for the BSFL treatment where it was slightly reduced (Table 7).

**TABLE 7:** Atlantic salmon performance, biometrics and blood metabolite profile when fed the experimental diets of the present study. As uneaten feed collection was not reliable in the 0FM0FO groups, total feed intake and FCR values are not calculated for this treatment.

	FMFO	TM	BSFM	PM	HM	0FM0FO	*p* value^a^
Fish number per tank	50	50	50	50	50	50	-
Start BW g	142 ± 1.5	142 ± 2.9	142 ± 0.2	141 ± 0.7	142 ± 0.5	141 ± 1.4	-
End BW g	475^c^ ±13.1	432^b^ ± 8.7	449^b^ ± 3.1	432^b^ ± 4.7	439^b^ ± 10.9	372^a^ ±9.7	0.000
BW increase g	333^c^ ±13.2	291^b^ ± 7.6	306^b^ ± 3.2	291^b^ ± 4.3	297^b^ ± 10.5	231^a^ ±9.6	0.000
TGC	4.16^c^ ±0.12	3.79^b^ ± 0.07	3.93^b^ ± 0.03	3.80^b^ ± 0.04	3.85^b^ ± 0.09	3.21^a^ ±0.10	0.000
FCR	0.74^a^ ±0.03	0.81^b^ ± 0.02	0.77^a^ ±0.03	0.74^a^ ±0.01	0.76^a^ ±0.02		0.017
Cumulative feed intake g fish^−1^	12,336 ± 862	11,842 ± 480	11,785 ± 604	10,793 ± 276	11,224 ± 470		0.065
D%	87.0 ^abc^ +0.6	86.3 ^abc^ +0.6	86.0^ab^ + 0.3	87.2^c^ +0.1	87.1 ^bc^ +0.5	85.8^a^ +0.2	0.007
CF	1.5^b^ + 0.04	1.49^b^ + 0.06	1.47^ab^ + 0.05	1.44^ab^ + 0.02	1.45^ab^ + 0	1.36^a^ +0.05	0.014
HSI	1.36^a^ +0.06	1.37^a^ +0.06	1.54^b^ + 0.02	1.45^ab^ + 0.07	1.34^a^ +0.03	1.38^ab^ + 0.09	0.014
Serum cortisol (nmol/L)	98.8 ± 38	93.5 ± 17	126.5 ± 74	117.0 ± 71	89.3 ± 37	182.4 ± 110	Ns
Serum K (mmol/L)	1.94 ± 1.25	0.91 ± 0.34	1.37 ± 0.79	1.54 ± 0.50	1.40 ± 0.74	0.71 ± 0.13	Ns
Serum glucose (mmol/L)	4.95 ^abc^ ±0.25	4.87^ab^ ± 0.38	5.54 ^cd^ ± 0.16	5.40 ^bc^ ±0.24	4.73^a^ ±0.34	6.13 ^d^ ± 0.51	0.003
Serum total protein (g/L)	48.7 ± 1.95	49.4 ± 2.93	48.6 ± 3.93	45.1 ± 4.29	48.9 ± 2.13	49.8 ± 2.63	ns
Serum cholesterol (mmol/L)	19.9 ± 0.87	19.2 ± 2.85	19.2 ± 1.68	15.1 ± 1.89	18.6 ± 2.09	16.7 ± 1.82	0.074
Serum ASAT U/L	778^c^ ±90	617 ^bc^ ±146	544^ab^ ± 175	559^ab^ ± 121	538^ab^ ± 67	308^a^ ±140	0.020
Serum ALAT U/L	7.4^b^ ± 0.85	5.2^a^ ±1.42	6.2^a^ ±2.40	5.1^a^ ±1.13	4.8^a^ ±0.18	3.4^a^ ±0.86	0.026
Serum CK U/L	36,509 ± 6,521	27,670 ± 6,215	17,281 ± 5,737	24,284 ± 10,715	21,489 ± 6,392	12,456 ± 8,351	ns

a Values in the same line with different small superscript letter are significantly different (*p* < 0.05) following Tukey post hoc test.

The total amino acid levels (66–67%) and profile of filet were similar in all treatments except for the BSFL treatment where fish filets contained significantly lower levels of His and total amino acids (63%) (Table 8). Last, though the levels of essential trace minerals varied significantly among the 6 test diets, in the filet they were very similar in all treatments except BSFL were filets contained lower levels of Zn (Table 8).

**TABLE 8:** NQC fillet nutritional quality of Atlantic salmon fed diets containing low trophic organism-based ingredients as FM and/or FO replacement, in terms of total lipids (% in freeze dried NQC fillet), fatty acids profile (% in B&D extract), protein, total amino acids and essential trace elements (% in freeze dried NQC fillet). The values are means of 3 values, representing the mean of each replicate group for each treatment ± standard variation.

	FMFO	TM	BSFM	PM	HM	0FM0FO	*p* value^a^
**Moisture wet%**	71.1 ± 0.56	71.1 ± 0.21	70.9 ± 0.56	71.8 ± 0.49	71.8 ± 0.1	71.9 ± 0.91	0.107
**Lipids (Bligh & Dyer)**	24.57^ab^ ± 1.21	24.73^ab^ ± 1.4	28.83^c^ ±2.55	24.73 ^abc^ ±3.25	23.8^ab^ ± 0.82	22.4^a^ ±1.76	0.037
**Protein**	74.23^ab^ ± 0.^75^	74.3^ab^ ± 1.21	69.93^a^ ±2.10	74.43^ab^ ± 2.67	75.2^b^ ± 0.50	75.33^b^ ± 2.05	0.023
**Moisture freeze dried%**	0.23 ± 0.4	0.17 ± 0.29	0.1 ± 0.17	0.17 ± 0.29	0 ± 0	0.2 ± 0.35	Ns
**Freeze drying factor**	0.288^ab^ ± 0.004	0.288^ab^ ± 0.005	0.290^b^ ± 0.005	0.280^ab^ ± 0.005	0.282^ab^ ± 0.001	0.279^a^ ±0.006	0.026
**C14:0**	3.27^c^ ±0.12	2.93^b^ ± 0.06	3.63 ^d^ ± 0.15	3.1 ^bc^ ±0.00	1.27^a^ ±0.06	1.43^a^ ±0.06	0.000
**C16:0**	11.20^a^ ±0.20	10.67^a^ ±0.21	11.13^a^ ±0.45	11.2^a^ ±0.26	12.50^b^ ± 0.00	12.2^b^ ± 0.20	0.000
**C18:0**	2.10^a^ ±0.10	2.23^a^ ±0.06	2.13^a^ ±0.06	2.2^a^ ±0.1	2.57^b^ ± 0.12	2.6^b^ ± 0.10	0.000
**C20:0**	0.20 ± 0.00	0.20 ± 0.00	0.17 ± 0.06	0.2 ± 0.00	0.2 ± 0.00	0.23 ± 0.06	Ns
**C22:0**	0.10 ± 0.00	0.10 ± 0.00	0.1 ± 0.00	0.1 ± 0.00	0.1 ± 0.00	0.10 ± 0.00	Ns
Saturated fatty acids	**16.87 ± 0.4**	**16.13 ± 0.23**	**17.17 ± 0.70**	**16.80 ± 0.35**	**16.63 ± 0.06**	**16.57 ± 0.25**	**0.099**
**C16:1n-7**	2.27^c^ ±0.06	2.13^c^ ±0.06	2.47 ^d^ ± 0.12	2.87^e^ ±0.06	1.07^a^ ±0.06	1.77^b^ ± 0.06	0.000
**C18:1 (n-9) + (n-7) + (n-5)**	24.87^ab^ ± 0.25	26.6^b^ ± 0.66	23.07^a^ ±1.06	24.17^a^ ±0.38	30.87^c^ ±0.15	30.27^c^ ±1.01	0.000
**C20:1 (n-9) + (n-7)**	7.33 ^d^ ± 0.06	6.67^c^ ±0.12	6.9^c^ ±0.17	6.67^c^ ±0.06	3.00^b^ ± 0.10	2.53^a^ ±0.06	0.000
**C22:1 (n-11) + (n-9) + (n-7)**	8.17^c^ ±0.12	7.37^b^ ± 0.21	8.03^c^ ±0.4	7.4^b^ ± 0.10	2.13^a^ ±0.06	1.67^a^ ±0.06	0.000
**C24:1 n-9**	0.50^b^ ± 0.00	0.47^b^ ± 0.06	0.47^b^ ± 0.06	0.50^b^ ± 0.00	0.30^a^ ±0.00	0.23^a^ ±0.06	0.000
Monounsaturated fatty acids	**43.13** ^**b**^ **± 0.21**	**43.23** ^**b**^ **± 0.95**	**40.93** ^**b**^ **± 1.60**	**41.60** ^**b**^ **± 0.53**	**37.37** ^**a**^ **±0.29**	**36.47** ^**a**^ **±1.11**	**0.000**
**C16:2 n-4**	0.10 ± 0.00	0.10 ± 0.00	0.1 ± 0.00	0.20 ± 0.00	0.00 ± 0.00	0.10 ± 0.00	ns
**C16:3 n-4**	0.07 ± 0.06	0.00 ± 0.00	0.07 ± 0.06	0.07 ± 0.06	0.00 ± 0.00	0.07 ± 0.06	ns
**C18:2 n-6**	9.60^a^ ±0.10	10.27^a^ ±0.21	9.2^a^ ±0.4	9.5^a^ ±0.17	12.27^b^ ± 0.21	12.1^b^ ± 0.87	0.000
**C18:3 n-6**	0.10 ± 0.00	0.17 ± 0.06	0.13 ± 0.06	0.13 ± 0.06	0.10 ± 0.00	0.13 ± 0.06	ns
**C20:2 n-6**	0.80^a^ ±0.00	0.77^a^ ±0.06	0.73^a^ ±0.06	0.73^a^ ±0.06	1.00^b^ ± 0.00	0.80^a^ ±0.00	0.000
**C20:3 n-6**	0.20^a^ ±0.00	0.30^b^ ± 0.00	0.30^b^ ± 0.00	0.30^b^ ± 0.00	0.23^a^ ±0.06	0.30^b^ ± 0.00	0.000
**C20:4 n-6**	0.10 ± 0.00	0.10 ± 0.00	0.10 ± 0.00	0.20 ± 0.00	0.10 ± 0.00	0.20 ± 0.00	ns
PUFA (n-6) fatty acids	**10.80** ^**a**^ **±0.10**	**11.60** ^**a**^ **±0.30**	**10.47** ^**a**^ **±0.50**	**10.87** ^**a**^ **±0.23**	**13.7 ** ^**b**^ **± 0.17**	**13.53 ** ^**b**^ **± 0.90**	**0.000**
**C18:3 n-3**	3.70^ab^ ± 0.00	4.90^c^ ±0.20	3.13^a^ ±0.15	3.87^b^ ± 0.15	7.57^a^ ±0.12	6.23 ^d^ ± 0.55	0.000
**C18:4 n-3**	0.83^b^ ± 0.06	0.90^b^ ± 0.00	0.83^b^ ± 0.06	0.87^b^ ± 0.06	0.43^a^ ±0.06	0.47^a^ ±0.06	0.000
**C20:3 n-3**	0.30 ± 0.00	0.30 ± 0.00	0.20 ± 0.00	0.30 ± 0.00	0.70 ± 0.00	0.40 ± 0.00	ns
**C20:4 n-3**	0.90 ^d^ ± 0.00	0.80^c^ ±0.00	0.90 ^d^ ± 0.00	0.80^c^ ±0.00	0.60^b^ ± 0.00	0.47^a^ ±0.06	0.000
**C20:5 n-3 (EPA)**	2.13^c^ ±0.06	1.77^b^ ± 0.12	2.00 ^bc^ ±0.10	2.10^c^ ±0.10	0.83^a^ ±0.06	0.80^a^ ±0.10	0.000
**C21:5 n-3**	0.10 ± 0.00	0.10 ± 0.00	0.1 ± 0.00	0.1 ± 0.00	0.00 ± 0.00	0.00 ± 0.00	ns
**C22:5 n-3**	0.93^b^ ± 0.06	0.80^b^ ± 0.00	0.9 ^bc^ ±0	0.97^c^ ±0.06	0.47^a^ ±0.06	0.47^a^ ±0.06	0.000
**C22:6 n-3 (DHA)**	6.00 ^bc^ ±0.10	5.50^ab^ ± 0.10	5.07^a^ ±0.23	6 ^bc^ ±0.36	7.93 ^d^ ± 0.25	6.47^c^ ±0.32	0.000
PUFA (n-3) fatty acids	**14.9** ^**b**^ **± 0.17**	**15.07** ^**b**^ **± 0.29**	**13.13** ^**a**^ **±0.50**	**15.00 ** ^**b**^ **± 0.52**	**18.53** ^**c**^ **±0.35**	**15.3** ^**b**^ **± 1.13**	**0.000**
**Total-PUFA fatty acids**	25.87^ab^ ± 0.21	26.77 ^bc^ ±0.57	23.77^a^ ±1.050	26.13^ab^ ± 0.45	32.23 ^d^ ± 0.51	29.00^c^ ±2.08	0.000
**Omega-6/omega-3 ratio**	0.73^a^ ±0.01	0.77 ^bc^ ±0.01	0.80^c^ ±0.01	0.72^a^ ±0.03	0.74^ab^ ± 0.01	0.88 ^d^ ± 0.010	0.000
**EPA ± DHA**	8.13^c^ ±0.15	7.27^ab^ ± 0.15	7.07^a^ ±0.32	8.10 ^bc^ ±0.46	8.77 ^d^ ± 0.25	7.27^ab^ ± 0.42	0.000
**Total identified fatty acids**	85.87 ± 0.75	86.13 ± 1.74	81.87 ± 3.35	84.53 ± 1.17	86.23 ± 0.78	82.03 ± 3.37	0.079
**Total unidentified fatty acids**	3.97^b^ ± 0.12	3.80^b^ ± 0.20	5.63^c^ ±0.38	4.17^b^ ± 0.15	2.97^a^ ±0.06	4.07^b^ ± 0.12	0.000
**Aspartic acid**	7.27 ± 0.12	7.17 ± 0.06	6.77 ± 0.25	7.03 ± 0.21	7.23 ± 0.06	7.20 ± 0.44	ns
**Glutamic acid**	10.3 ± 0.36	10.30 ± 0.30	9.7 ± 0.36	10.27 ± 0.46	10.17 ± 0.38	10.40 ± 0.20	ns
**Serine**	2.87 ± 0.06	2.93 ± 0.06	2.8 ± 0.00	2.87 ± 0.06	2.90 ± 0.00	2.93 ± 0.15	ns
**Glycine**	3.60 ± 0.20	3.60 ± 0.10	3.47 ± 0.15	3.63 ± 0.15	3.57 ± 0.06	3.63 ± 0.06	ns
**Histidine**	2.33^b^ ± 0.15	2.27^b^ ± 0.06	1.87^a^ ±0.06	2.23^b^ ± 0.06	2.27^b^ ± 0.06	2.20^b^ ± 0.10	0.000
**Arginine**	4.1 ± 0.10	4.07 ± 0.12	3.8 ± 0.17	4.00 ± 0.30	4.00 ± 0.00	4.00 ± 0.10	ns
**Threonine**	3.33 ± 0.06	3.37 ± 0.06	3.13 ± 0.06	3.23 ± 0.21	3.33 ± 0.06	3.30 ± 0.20	ns
**Alanine**	3.93 ± 0.40	3.80 ± 0.40	3.7 ± 0.30	4.00 ± 0.30	4.27 ± 0.51	3.93 ± 0.49	ns
**Proline**	2.33 ± 0.15	2.33 ± 0.06	2.23 ± 0.15	2.43 ± 0.15	2.60 ± 0.36	2.37 ± 0.29	ns
**Tyrosine**	2.50 ± 0.00	2.47 ± 0.15	2.33 ± 0.15	2.47 ± 0.06	2.50 ± 0.17	2.43 ± 0.15	ns
**Valine**	3.87 ± 0.15	3.87 ± 0.21	3.67 ± 0.21	3.80 ± 0.20	3.90 ± 0.10	3.90 ± 0.20	ns
**Methionine**	2.3 ± 0.00	2.30 ± 0.10	2.17 ± 0.06	2.30 ± 0.10	2.23 ± 0.29	2.30 ± 0.17	ns
**Isoleucine**	3.37 ± 0.12	3.33 ± 0.15	3.13 ± 0.15	3.27 ± 0.21	3.30 ± 0.10	3.30 ± 0.10	ns
**Leucine**	5.57 ± 0.06	5.43 ± 0.15	5.17 ± 0.12	5.43 ± 0.21	5.53 ± 0.06	5.50 ± 0.26	ns
**Phenylalanine**	2.93 ± 0.06	2.87 ± 0.06	2.77 ± 0.06	2.87 ± 0.15	2.97 ± 0.06	2.93 ± 0.23	ns
**Lysine**	6.80 ± 0.17	6.63 ± 0.25	6.40 ± 0.17	6.67 ± 0.31	6.77 ± 0.12	6.63 ± 0.32	ns
**Total amino acids**	67.40 ± 1.15	66.73 ± 1.47	63.1 ± 1.68	66.5 ± 2.66	67.53 ± 0.70	66.97 ± 3.32	ns
**Fe**	9.47 ± 0.21	9.70 ± 1.21	11.67 ± 2.08	11.33 ± 0.58	10.80 ± 1.31	11.33 ± 0.58	ns
**Zn**	15.67ab ± 0.58	16.67^b^ ± 0.58	14.67^a^ ±0.58	16.67^b^ ± 0.58	16.00^ab^ ± 0.00	17.00^b^ ± 1.00	0.006
**Se**	0.70 ± 0.00	0.67 ± 0.06	0.60 ± 0.00	0.63 ± 0.06	0.63 ± 0.06	0.63 ± 0.06	ns
**Mn**	0.37 ± 0.12	0.30 ± 0.00	0.33 ± 0.06	0.30 ± 0.00	0.37 ± 0.06	0.37 ± 0.06	ns

a Values in the same line with different small superscript letter are significantly different (*p* < 0.05) following Tukey post hoc test.

### Skin Composition and Histology

The amino acid composition of the skin was similar in fish from all treatments with typical profile of a collagen rich tissue, containing relatively high levels of the dispensable amino acids Gly, Hyp, and Pro and lower relative levels of non-dispensable amino acids (Table 9). Atlantic salmon skin was rich in Zn, followed by Mn and Fe, and contained also higher levels Se but lower levels of Cu as compared to the fillets (Tables 8 and 9). Minerals are involved in a great number of reactions, i. e, related to their antioxidant function. For instance, superoxide dismutase (SOD), part of the cellular antioxidant defence, are associated with essential trace metals. In the skin, there are found SODs associated with Cu-Zn, Fe and Mn, and their activities are found to be higher in the dark than in the light parts of skin (Nakano et al., 1993) which explain their mineralisation pattern. As for amino acids, and as in filet, the levels of essential trace minerals in the skin were very similar among fish from different dietary treatments, unlike the large differences among the diets, introduced by the great raw material differences in this respect. Nevertheless, there was some tendencies and significant but small differences in Se and Mn levels in skin. The skin of Atlantic salmon in PM and 0FM0FO treatments had lower levels Mn than fish in the FMFO and BSFL treatments. Moreover, there was analysed less Se in the skin of fish in BSFL and 0FM0FO treatments as compared to the rest.

**TABLE 9:** Skin moisture, amino acid profile (% in freeze dried skin sample) and trace mineral content (mg kg^−1^ freeze dried skin sample) in Atlantic salmon fed diets containing low trophic organism-based ingredients as FM and/or FO replacement. The values for moisture are in wet samples whereas those for amino acids and minerals are in freeze dried samples. Values are means of 3 values, representing the mean of each replicate group for each treatment ± standard variation.

	FMFO	TM	BSFM	PM	HM	0FM0FO	*p* value^a^
**Moisture wet%**	61.4 ± 1.9	60.2 ± 4.3	63.5 ± 1.08	62 ± 0.7	62.5 ± 0.63	63.1 ± 0.97	Ns
**Freeze drying factor**	0.386 ± 0.019	0.398 ± 0.043	0.365 ± 0.011	0.38 ± 0.007	0.375 ± 0.006	0.369 ± 0.01	Ns
**Aspartic acid**	4.97 ± 0.23	5.10 ± 0.10	5.03 ± 0.23	5.13 ± 0.21	5.00 ± 0.10	5.07 ± 0.12	Ns
**Glutamic acid**	7.37 ± 0.32	7.43 ± 0.15	7.3 ± 0.44	7.5 ± 0.35	7.27 ± 0.21	7.47 ± 0.21	Ns
**Hydroxyproline**	3.97 ± 0.57	4.27 ± 0.32	4.10 ± 0.44	4.37 ± 0.49	4.07 ± 0.21	3.97 ± 0.21	Ns
**Serine**	3.10 ± 0.10	3.17 ± 0.15	3.200 ± 0.1	3.23 ± 0.06	3.17 ± 0.06	3.17 ± 0.06	Ns
**Glycine**	13.67 ± 0.86	14.00 ± 0.50	13.33 ± 1.01	13.87 ± 0.78	13.30 ± 0.61	13.57 ± 0.38	Ns
**Histidine**	1.63 ± 0.15	1.63 ± 0.06	1.53 ± 0.15	1.67 ± 0.15	1.53 ± 0.06	1.60 ± 0.00	Ns
**Arginine**	4.8 ± 0.2	4.9 ± 0.20	4.70 ± 0.35	4.87 ± 0.25	4.73 ± 0.15	4.8 ± 0.17	Ns
**Threonine**	2.1 ± 0.00	2.13 ± 0.06	2.10 ± 0.10	2.07 ± 0.06	2.07 ± 0.06	2.13 ± 0.06	Ns
**Alanine**	5.17 ± 0.35	5.43 ± 0.38	5.30 ± 0.61	5.40 ± 0.20	5.20 ± 0.40	5.33 ± 0.15	Ns
**Proline**	6.33 ± 0.38	6.83 ± 0.23	6.60 ± 0.62	6.57 ± 0.23	6.40 ± 0.36	6.37 ± 0.35	Ns
**Tyrosine**	1.13 ± 0.06	1.20 ± 0.00	1.23 ± 0.06	1.17 ± 0.06	1.13 ± 0.06	1.17 ± 0.06	Ns
**Valine**	2.00 ± 0.00	2.00 ± 0.00	2.03 ± 0.06	2.03 ± 0.06	2.00 ± 0.00	2.00 ± 0.10	Ns
**Methionine**	2.17 ± 0.06	2.13 ± 0.06	2.10 ± 0.10	2.17 ± 0.06	2.10 ± 0.10	2.10 ± 0.10	ns
**Isoleucine**	1.40 ± 0.00	1.37 ± 0.06	1.43 ± 0.06	1.4 ± 0.00	1.4 ± 0.00	1.43 ± 0.06	ns
**Leucine**	2.60 ± 0.10	2.57 ± 0.06	2.63 ± 0.06	2.63 ± 0.06	2.63 ± 0.06	2.60 ± 0.10	ns
**Phenylalanine**	1.83 ± 0.12	1.83 ± 0.06	1.83 ± 0.06	1.87 ± 0.06	1.80 ± 0.00	1.83 ± 0.06	ns
**Lysine**	2.77 ± 0.21	2.93 ± 0.12	3.00 ± 0.26	2.97 ± 0.23	2.87 ± 0.15	2.90 ± 0.3	ns
Total amino acids	**67 ± 3.04**	**68.93 ± 1.57**	**67.47 ± 4.3**	**68.9 ± 3.16**	**66.67 ± 1.8**	**67.5 ± 2.25**	**ns**
**Cu**	1.37 ± 0.23	1.57 ± 0.21	1.5 ± 0.17	1.87 ± 0.46	1.60 ± 0.10	1.53 ± 0.15	ns
**Fe**	16.5 ± 3.54	16.00 ± 2.00	16.67 ± 2.52	21.33 ± 7.77	16.33 ± 1.53	16.67 ± 2.52	ns
**Zn**	116.67 ± 11.55	116.67 ± 15.28	110 ± 20	133.33 ± 11.55	120.00 ± 10.00	126.67 ± 11.55	ns
**Se**	1.03 ± 0.06	0.97 ± 0.06	0.90 ± 0.00	0.97 ± 0.06	1.03 ± 0.06	0.90 ± 0.10	0.071
**Mn**	17.33^b^ ± 0.58	15.33^ab^ ± 1.53	16.67^b^ ± 1.15	13.33^a^ ±0.58	15.00^ab^ ± 1.00	13.00^a^ ±0.00	0.001

a Values in the same line with different small superscript letter are significantly different (*p* < 0.05) following Tukey post hoc test.

**FIGURE 2:**
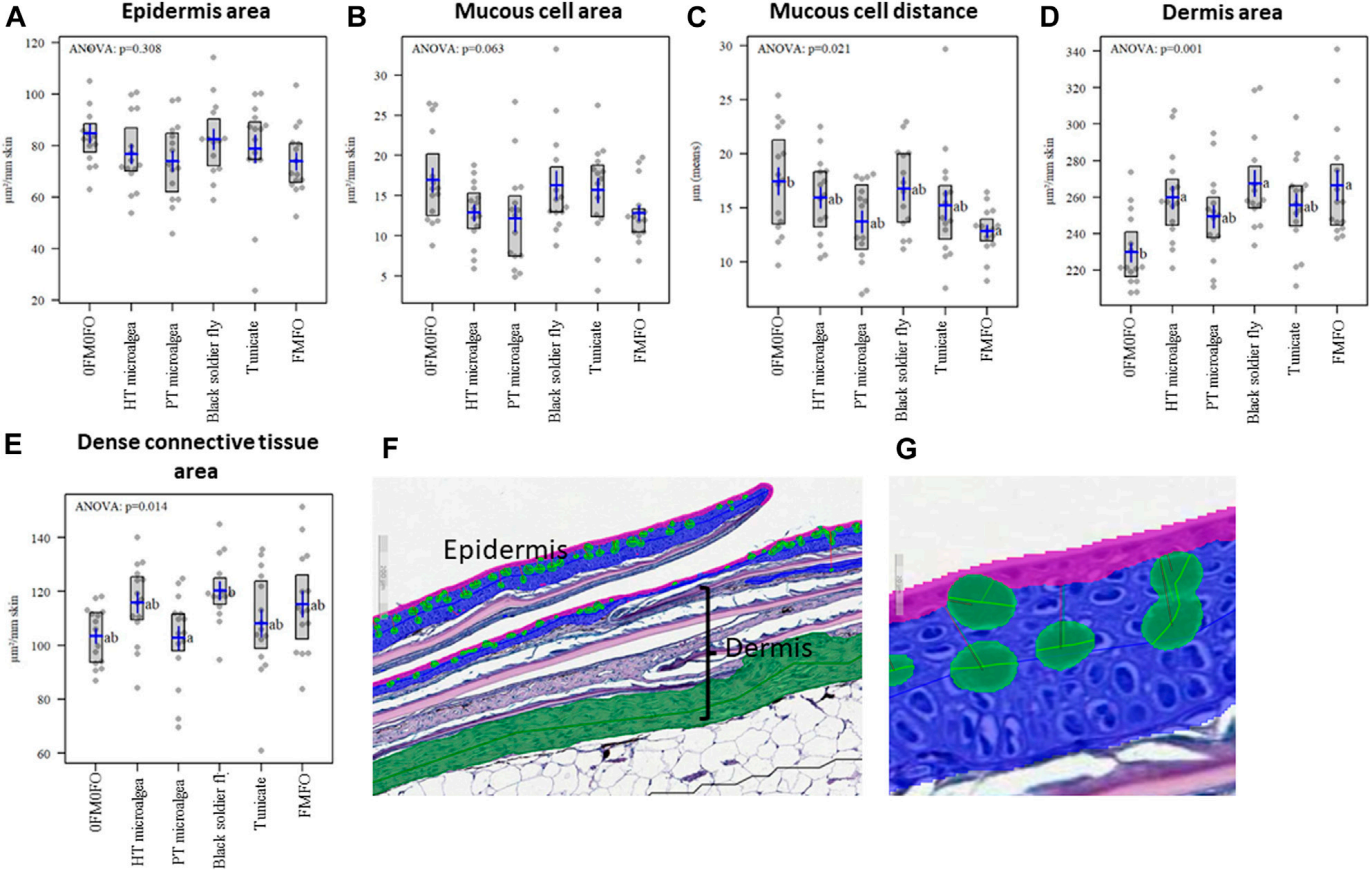
Aiforia® results for **(A)** Epidermis area, **(B)** Mucous cell area, **(C)** Mucous cell distance, **(D)** Dermis area, and **(E)** Dense connective tissue area in histological samples of skin **(F,G)** from Atlantic salmon in the experimental treatments of the current study.

Histological quantification of skin tissues showed a thinner dermis for the 0FM0FO fish, which can be because those fish were the smallest among all trial treatments. The dense connective tissue of the skin was thicker for the BSFL as compared to the PM group, and these two groups where those that differed from the others in terms of their skin content in Se and Mn. The scale areas followed a similar pattern as dermis with lowest values for the 0FM0FO fish (Figure 2). There was no effect on the epidermal area or number and area of mucous cells, and the epidermis of the fish studied looked healthy.

## Conclusion

Our study shows that *S. limacinum* biomass, disrupted *Phaeodactylum tricornutum* biomass, black soldier fly larvae (*Hermetia illucens*) meal, and tunicate (*Ciona intestinalis*) meal are well accepted raw materials and can sustain a healthy Atlantic salmon with high growth rate and low FCR fed low or no FM and FO in the diet. Nevertheless, the performance of fish fed diets containing high quality fresh and organic FM and FO was superior to each one, and even more when all four test raw materials were combined. The performance of novel raw materials as ingredients is aquafeeds may vary according to the marine raw materials they are compared to, ranging from performing equally or better than an average quality FM and FO to performing inferiorly to high quality fresh FM and FO. Besides being sources of proteins and lipids, the studied ingredients can be used as rich natural sources of limiting non-dispensable amino acids, trace minerals and vitamins. However, the degree of stability and availability of these non-dispensable nutrients in extruded diets for fish should be established and safeguarded. Possible physiological effects of high Mn level in black soldier fly larvae meal are worth further investigation. Fish filet lipids, amino acid and trace mineral profile and level as well as the profile and levels of amino acids and trace mineral in the skin were conservative to a great degree unlike the compositional differences among the experimental diets.

## Data Availability

The original contributions presented in the study are included in the article/Supplementary Material, further inquiries can be directed to the corresponding author.
