# Supplementary material for: Atlantic Salmon (Salmo salar) Performance Fed Low Trophic Ingredients in a Fish Meal and Fish Oil Free Diet
Source: Front Physiol. 2022 Jun 8;13:884740. doi: 10.3389/fphys.2022.884740 (PMC9214214; doi:10.3389/fphys.2022.884740)
Supplement: Supplementary file 1 [file DataSheet1.docx]

Supplementary Table 1. Proximate composition and total amino acid profile of organic FM and the test ingredients used in our study, expressed in g 100g^-1^ sample. Analyses are performed in duplicate samples per value presented. Empty cells indicating missing analytical values.

| Analysed parameter | Organic FM | Tunicate meal | Black soldier fly larvae meal | *Phaeodactylum tricornutum* | *Schizochytrium limacinum* |
| --- | --- | --- | --- | --- | --- |
| Moisture |  | 5.11 | 5.0 | 2.8 | 1.3 |
| Crude protein Kjeldahl (N*6.25) | 70.9 | 40.8 | 59.8 | 43.4 | 12.4 |
| Water soluble protein | 16.8 |  | 13.50 | 15.73 | 7.19 |
| Ash |  | 26.5 | 7.1-9.0 | 16.7 | 4.4 |
| Starch |  |  |  | 0.2 | 1.2 |
| Salt |  |  |  | 3.425 |  |
| Aspartic acid | 6.7 | 4.23 | 5.6 | 3.8 | 0.87 |
| Glutamic acid | 9.4 | 4.84 | 6.1 | 5.65 | 1.23 |
| Hydroxyproline | 0.67 | <0.05 | 0 | 0.1 | 0 |
| Serine | 3 | 1.98 | 2.4 | 1.7 | 0.55 |
| Glycine | 4.6 | 2.39 | 3.3 | 2.1 | 0.47 |
| Histidine | 1.8 | 0.863 | 1.9 | 0.63 | 0.21 |
| Arginine | 4.4 | 2.54 | 2.7 | 2.25 | 0.52 |
| Threonine | 3.1 | 2.06 | 2.2 | 1.8 | 0.40 |
| Alanine | 4.5 | 1.84 | 3.8 | 2.85 | 0.55 |
| Proline | 2.9 | 1.53 | 3.2 | 1.95 | 0.32 |
| Tyrosine | 2.4 | 1.52 | 3.6 | 1.15 | 0.29 |
| Valine | 3.6 | 1.98 | 3.4 | 2.2 | 0.52 |
| Methionine | 2.3 | 0.772 | 1.1 | 0.865 | 0.21 |
| Isoleucine | 3 | 1.68 | 2.5 | 1.85 | 0.42 |
| Leucine | 5.3 | 2.62 | 3.9 | 3 | 0.67 |
| Phenylalanine | 2.8 | 1.58 | 2.4 | 2.05 | 0.41 |
| Lysine | 5.8 | 2.48 | 3.3 | 2.3 | 0.78 |
| Cysteine/Cystine | 0.67 | 0.882 | 0.45 | 0.55 | 0.22 |
| Tryptophan | 0.84 | 0.471 | 0.96 | 0.63 | 0.16 |
| **Total amino acids** | **67.8** | **36.3** | **52.8** | **37.4** | **8.8** |
| **Non-dispensable Amino Acids (NDAA)** | **32.9** | **17.0** | **24.4** | **17.6** | **4.3** |
| **NDAA/DAA** | **0.9** | **0.9** | **0.9** | **0.9** | **1.0** |

Supplementary Table 2. Free amino acid profile of organic fishmeal and the test ingredients used in our study, expressed in g 100g^-1^ sample. Analyses are performed in duplicate samples per value presented.

| Analysed parameter | Organic FM | Tunicate meal | Black soldier fly larvae meal | *Phaeodactylum tricornutum* | *Schizochytrium limacinum* |
| --- | --- | --- | --- | --- | --- |
| Creatinine | 0.52 | <0.01 | <0.01 | 0 | 0.095 |
| Asparagine acid | 0.02 | 0.01 | 0.04 | 0.1 | 0.017 |
| Glutamic acid | 0.12 | 0.07 | 0.11 | 1.245 | 0.056 |
| Hydroxyproline | <0.01 | <0.01 | <0.01 | 0 | 0.001 |
| Serine | 0.02 | <0.01 | 0.03 | 0.115 | 0.071 |
| Asparagine | 0.01 | <0.01 | 0.03 | 0.25 | 0.049 |
| Glycine | 0.09 | 0.09 | 0.08 | 0.485 | 0.034 |
| Glutamine | 0.01 | 0.06 | 0.08 | 0.155 | 0.11 |
| 3-amino-propionic acid | 0.09 | <0.01 | 0.01 | 0.01 | <0.001 |
| Taurine | 0.47 | 0.16 | 0.05 | 0.06 | 0.018 |
| Histidine | <0.01 | <0.01 | 0.07 | 0.025 | 0.013 |
| 4-amino-butanoic acid | <0.01 | <0.01 | 0.02 | 0 | 0.059 |
| Citrulline | 0.01 | 0.01 | 0.03 | 0.01 | <0.001 |
| Threonine | 0.04 | 0.01 | 0.06 | 0.125 | 0.047 |
| Alanine | 0.16 | 0.03 | 0.26 | 0.85 | 0.084 |
| Carnosine | <0.01 | <0.01 | <0.01 | 0 | 0.016 |
| Arginine | 0.04 | 0.02 | 0.27 | 0.32 | 0.067 |
| Proline | 0.01 | 0.02 | 0.27 | 0.955 | 0.041 |
| Tyrosine | 0.03 | 0.01 | 0.23 | 0.1 | 0.079 |
| Valine | 0.06 | 0.01 | 0.06 | 0.14 | 0.097 |
| Methionine | 0.02 | 0.01 | <0.01 | 0.07 | 0.047 |
| Cystine | <0.01 | <0.01 | <0.01 | 0 | <0.001 |
| Isoleucine | 0.05 | 0.01 | 0.03 | 0.1 | 0.074 |
| Leucine | 0.1 | 0.02 | 0.04 | 0.15 | 0.157 |
| Phenylalanine | 0.04 | 0.01 | 0.02 | 0.09 | 0.088 |
| Tryptophan | 0.01 | <0.01 | 0.07 | 0.04 | 0.027 |
| Ornithine | 0.01 | <0.01 | <0.01 | 0.435 | 0.002 |
| Lysine | 0.05 | 0.01 | 0.04 | 0.365 | 0.054 |
| Total free amino acids | 1.98 | 0.56 | 1.9 | 6.195 | 1.403 |

Supplementary Table 3. Water soluble protein peptide size distribution in organic fishmeal and the test ingredients used in our study, expressed in g 100g^-1^ water soluble protein. Analyses are performed in duplicate samples per value presented.

| Peptide size (in Da) | Organic FM | Tunicate meal | Black Soldier fly larvae meal | *Phaeodactylum tricornutum* | *Schizochytrium limacinum* |
| --- | --- | --- | --- | --- | --- |
| >20000 | 3.20 | 2.00 | 0.40 | 1.93 | 0.40 |
| 15000-20000 | 3.50 | 0.90 | 0.50 | 0.20 | <0,1 |
| 10000-15000 | 6.50 | 1.90 | 2.70 | 0.33 | 0.10 |
| 8000-10000 | 3.80 | 1.20 | 5.10 | 0.23 | 0.30 |
| 6000-8000 | 4.90 | 2.00 | 8.10 | 0.73 | 1.00 |
| 4000-8000 | 6.00 | 3.90 | 7.30 | 1.70 | 2.80 |
| 2000-4000 | 6.40 | 8.80 | 7.60 | 3.60 | 5.90 |
| 1000-2000 | 3.30 | 10.00 | 5.30 | 4.70 | 7.90 |
| 500-1000 | 2.20 | 11.00 | 4.80 | 6.37 | 10.50 |
| 200-500 | 4.50 | 14.40 | 8.60 | 8.37 | 15.50 |
| <200 | 55.70 | 43.80 | 49.70 | 71.77 | 55.70 |

Supplementary Table 4. Nucleotide composition of organic fishmeal and the test ingredients used in our study. Analyses are performed in duplicate samples per value presented. Are given as mg kg-1 sample.

| Analysed parameter | Organic FM | Black soldier fly larvae meal | *Phaeodactylum tricornutum* | *Schizochytrium limacinum* |
| --- | --- | --- | --- | --- |
| Hypoxanthine | 4860 | 340 | 500 | 490 |
| IMP | 1680 | 420 | 100 | <100 |
| Inosine | 2410 | 500 | 1530 | 540 |
| AMP | 390 | 1470 | 490 | 710 |
| ADP | <100 | 170 | 190 | <100 |
| ATP | <100 | <100 | <100 | <100 |
| K-value nucleotides | 88.3 | 43 | 83.6 | 71.6 |

Supplementary Table 5. Freshness, and processing quality indicators of organic fishmeal and the test ingredients used in our study. Analyses are performed in duplicate samples per value presented.

| Analysed parameter | Unit | Organic FM | Black Soldier fly larvae meal | *Phaeodactylum tricornutum* | *Schizochytrium limacinum* |
| --- | --- | --- | --- | --- | --- |
| Putrescine | mg/kg | 190 | 30 | <20 | 440 |
| Cadaverine | mg/kg | 360 | <20 | <20 | <20 |
| Histamine | mg/kg | 31 | <20 | <20 | <20 |
| Aerobic microorganisms | KDE/g | 230 | 410000 | 1600 | 460 |
| coliform bacteria at 37ºC | KDE/g |  |  | 100 |  |
| *E. coli* | KDE/g |  |  | <10 |  |
| Anaerobic sulphite reducing bacteria | KDE/g |  |  | <10 |  |
| *Clostridium perfingens* | KDE/g |  |  | <10 |  |
| Presumed *Bacillus cereus* (non-accredited) | KDE/g |  |  | <10 |  |
| Mould | KDE/g |  |  | 500 |  |
| Yeast | KDE/g |  |  | 300 |  |

Supplementary Table 6. Lipid content and fatty acid profile of organic fishmeal and the test ingredients used in our study. Analyses are performed in duplicate samples per value presented. Fatty acid amounts are given as g 100 g^-1^ Bligh & Dyer extract. Empty cells concerning the fatty acids profile of the ingredients indicate non-detectable or non-quantifiable amounts.

| Analysed parameter | Organic FM | Tunicate meal | Black Soldier fly larvae meal | *Phaeodactylum tricornutum* | *Schizochytrium limacinum* |
| --- | --- | --- | --- | --- | --- |
| Fat (Bligh & Dyer 1959)% | 11.1 |  | 10.2 | 16.0 | 57.5 |
| Fat (EU)% | 10.3 | 3.14 |  | 13.0 |  |
| Gross energy Kj/g |  |  |  | 19.48 |  |
| 14:0 | 4.5 | 0.4 | 6.0 | 2.9 | 4.2 |
| 15:0 |  | 0.21 |  |  | 2.2 |
| 16:0 | 11.9 | 2.65 | 11 | 7.3 | 52.9 |
| 17:0 |  | 0.19 |  |  | 0.7 |
| 18:0 | 1.6 | 0.76 | 1.8 | 0.2 | 1.5 |
| 20:0 | 0.1 | 0.24 | 0.5 |  | 0.2 |
| 22:0 | 0.1 | 0.09 | 0.1 |  | 0.2 |
| 24:0 |  | 0.02 |  |  |  |
| 16:1 n-7 | 2.5 | 0.57 | 1.4 | 9.1 | 0.1 |
| 18:1 (n-9)±(n-7)±(n-5) | 9.8 | 1.47 | 10.9 | 1.65 | 0.2 |
| 20:1 (n-9)±(n-7) | 8.5 | 0.09 | 0.4 |  | 0.1 |
| 22:1 (n-11)±(n-9)±(n-7) | 12.3 | 0.14 | 0.1 |  |  |
| 24:1 n-9 | 0.8 |  |  | 0.05 |  |
| 16:2 n-4 | 0.2 |  |  | 3.9 |  |
| 16:3 n-4 | 0.2 |  |  | 3.45 |  |
| 18:2 n-6 | 1.4 | 0.474 | 13.8 | 1.6 | 0.1 |
| 18:3 n-6 | 0.1 |  | 0.1 | 0.3 |  |
| 20:2 n-6 | 0.2 | 0.05 |  |  |  |
| 20:3 n-6 | 0.1 | 0.02 |  | 0.05 | 0.1 |
| 20:4 n-6 | 0.4 | 0.83 |  | 2.75 | 0.1 |
| 22:5 n-6 |  | 0.09 |  |  | 5.6 |
| 16:4 n-3 |  |  |  | 0.1 |  |
| 18:3 n-3 | 0.8 | 0.24 | 0.9 | 0.4 |  |
| 18:4 n-3 | 1.9 | 0.43 | 0.1 | 0.3 | 0.1 |
| 20:3 n-3 | 0.1 | 0.07 |  |  |  |
| 20:4 n-3 | 0.5 | 0.14 |  | 0.1 | 0.3 |
| 20:5 n-3 (EPA) | 5.8 | 5.40 |  | 14.2 | 0.4 |
| 21:5 n-3 | 0.2 |  |  |  |  |
| 22:5 n-3 | 0.9 | 0.21 |  |  | 0.1 |
| 22:6 n-3 (DHA) | 12.8 | 4.08 | 0.1 | 0.45 | 26.7 |
| Sum saturated fatty acids | 18.2 | 4.62 | 19.4 | 10.35 | 61.9 |
| Sum monoenoic fatty acids | 33.9 | 2.30 | 12.8 | 19.8 | 0.4 |
| Sum PUFA (n-6) fatty acids | 2.2 | 1.52 | 13.9 | 4.7 | 5.8 |
| Sum PUFA (n-3) fatty acids | 23 | 10.52 | 1.1 | 15.55 | 27.6 |
| Sum total-PUFA fatty acids | 25.6 | 12.04 | 15 | 27.6 | 33.4 |
| omega-6/omega-3 ratio | 0.09 | 0.14 | 12.41 | 0.30 | 0.21 |
| Sum EPA ± DHA | 18.6 | 9.48 | 0.1 | 14.65 | 27.1 |
| Sum identified fatty acids | 77.7 | 23.7 | 47.2 | 57.75 | 95.8 |
| Sum unidentified fatty acids | 4.6 | 76.3 | 36.2 | 9.3 | 4.2 |

Supplementary Table 7. Lipid class composition and oxidation status of organic fishmeal and the test ingredient lipids used in our study. Analyses are performed in duplicate samples per value presented. Lipid class amounts are given as g 100 g^-1^ Bligh & Dyer extract. Empty cells indicate missing analytical values.

| **Analysed parameter** | Organic FM | Tunicate meal | Black soldier fly larvae meal | *Phaeodactylum tricornutum* | *Schizochytrium limacinum* |
| --- | --- | --- | --- | --- | --- |
| Triacylglycerol | 59 |  | 63 | 6 | 97 |
| Diacylglycerol | <0.5 |  | 0.8 | 0 | 0.6 |
| Monoacylglycerol | <1 |  | <1 | 0 | <1 |
| Free fatty acids | 2.5 |  | 2.2 | 22.5-58.67 | 2-2.60 |
| Cholesterol | 1.4 |  | 0.5 | 0 | <0.5 |
| Cholesterol esters | <0.5 |  | <0.5 | 1.5 | <0.5 |
| Phosphatidyl ethanolamine | 5.4 |  | 4.8 | 5.4 | 1.4 |
| Phosphatidyl inositol | <1 |  | <1 | 0 | <1 |
| Phosphatidyl serine | <1 |  | <1 | 0 | <1 |
| Phosphatidyl choline | 18 |  | 11 | 0 | <1 |
| lyso-phosphatidyl choline | <0.5 |  | 1 | 0.85 | 1.2 |
| total polar lipids | 23.4 |  | 16.8 | 6.2 | 2.6 |
| Total neutral lipids | 62.9 |  | 66.5 | 29.75 | 100.1 |
| Total lipids | 86.3 |  | 83.3 | 35.95 | 102.8 |
| Tripalmitin |  |  |  |  | 32 |
| TBARS mg malonaldehyde/kg |  |  |  | 10.25 |  |

Supplementary Table 8. Vitamin, macro- and micro-mineral content of organic fishmeal and the test ingredient lipids used in our study. Analyses are performed in duplicate samples per value presented. Amounts are given in mg kg^-1^ unless otherwise indicated. Empty cells indicate missing analytical values.

| Analysed parameter | Organic FM | Tunicate meal | Black soldier fly larvae meal | *Phaeodactylum tricornutum* | *Schizochytrium limacinum* |
| --- | --- | --- | --- | --- | --- |
| Vit A (retinol) | 5.47 | <0.21 | <0.21 | <0.21 | 1.23 |
| Vit D3 | 0.0534 | <0.0025 | <0.0025 | <0.0025 | <0.0025 |
| Vit K1 μg/kg |  | 50.3 |  | 31.9 |  |
| Vit K2 μg/kg | <8 |  | <8 |  | <8 |
| alpha Tocopherol | 10.1 | 6.6 |  | 111 | <0.8 |
| beta Tocopherol | <5 |  |  | 26.2 |  |
| gamma Tocopherol | 29.3 |  |  | 12.4 |  |
| delta Tocopherol | 16.3 |  |  | 6.47 |  |
| Vitamin E (sum tocopherols) | 55.7 |  | 23.3 | 156.07 |  |
| Vitamin C (Ascorbic acid) |  |  |  | 1050 |  |
| P% |  | 0.44 | 1-1.1 | 0.355 | 0.21 |
| soluble P% |  |  | 0.695 | 0.21 | 0.2 |
| Ca% |  | 1 |  |  |  |
| Na% |  | 6.4 |  |  |  |
| Fe | 350 | 2200 | 220 | 190 |  |
| Mn | 5.7 | 71 | 350 | 120 |  |
| Cu | 4.1 | 14 | 14 | 3.3 |  |
| Se | 2.3 | 3.7 | 0.3 | <0.2 |  |
| Zn | 65 | 120 | 120 | 20 |  |

Supplementary Table 9. Maximum levels of undesirable substances in feed materials made from fish or other aquatic animals and in complete feed for fish, according to Directive 2002/32/EC and Regulation (EC) No 1881/2006, with amendments, and the respective levels in the feed raw materials used in this study. Analyses are performed in single samples per value presented. Empty cells indicate missing analytical values.

| Analysed parameter | Organic FM | Tunicate meal | Black soldier fly larval meal | *Phaeodactylum tricornutum* | *Schizochytrium limacinum* | *Maximum levels: Feed material made from fish* | *Maximum levels: Complete feed for fish* |
| --- | --- | --- | --- | --- | --- | --- | --- |
| Aldrin μg/kg | <0.1 |  | <0.1 | <0.1 | 0.0642 | *10* | *10* |
| Dioxins (Total PCDD/PCDF) pg/g (TEQ WHO 2005)* | 0.165 |  | 0.0388 | 0.0618 | 0.103 | *1.25* | *0.75* |
| Sum PCDD/PCDF & WHO-PCB pg/g (TEQ WHO 2005) | 0.304 |  | 0.103 | 0.099 | 0.373 |  |  |
| Non-DL-PCBs (''6 marker PCBs'') ng/g | 1.75 |  | 0.375 | 0.464 | <0.1 | *30* | *40* |
| Sum DDT-, TDE- & DDE-isomers μg/kg | <0.3 |  | <0.6 | <0.6 | <0.6 | *50* | *50* |
| Sum identified PAH 4 μg/kg | <2 |  | <2 | <2 | 1 | *-* | *-* |
| Hg mg/kg | 0.083 |  | <0.005 | 0.005 | <0.005 | *0.5* | *0.2* |
| Pb mg/kg | 0.05 | 1.8 | 0.18 | 0.05 | <0.05 | *10* | *5* |
| Cd mg/kg | 0.18 | 0.15 | 0.57 | <0.01 | <0.01 | *2* | *1* |
| As mg/kg | 3.4 |  | <0.1 | 0.2 | <0.1 | *2* | *10* |

*World Health Organization 2005 toxicity equivalent (Van den Berg et al., 2006).
